# Distribution Types of Lichens in Hungary That Indicate Changing Environmental Conditions

**DOI:** 10.3390/jof8060600

**Published:** 2022-06-03

**Authors:** Edit Farkas, Nóra Varga, Katalin Veres, Gábor Matus, Mónika Sinigla, László Lőkös

**Affiliations:** 1 Institute of Ecology and Botany, Centre for Ecological Research, H-2163 Vácrátót, Hungary; varga.nora@ecolres.hu (N.V.); veres.katalin@ecolres.hu (K.V.); 2 Department of Botany, Faculty of Science and Technology, University of Debrecen, H-4010 Debrecen, Hungary; matus.gabor@science.unideb.hu; 3 Bakony Museum of the Hungarian Natural History Museum, H-8420 Zirc, Hungary; monikasinigla@gmail.com; 4 Department of Botany, Hungarian Natural History Museum, H-1431 Budapest, Hungary; lokos.laszlo@nhmus.hu

**Keywords:** acidofrequent, air pollution bioindication, biodiversity, climate, environmental changes, land use, nitrofrequent, rapidly spreading, substrate, time scale

## Abstract

**Simple Summary:**

As the occurrences of lichens are strongly correlated to background environmental conditions (e.g., air pollution, global warming), the analysis of their distribution has a great value for bioindication. Distribution data are originating from earlier herbarium collections, recent field and literature studies. The distribution analyses in lichen species with different ecological requirements allowed comparisons and showed clear trends. Five distribution types were introduced—presented by characteristic examples—according to lichen distribution maps prepared in different periods of time (representing changing environmental conditions): (1) species of decreasing occurrences by time (acidic pollution sensitive species), (2) species with no or few former records but with increasing occurrences in recent decades (sub-Mediterranean species), (3) species with increasing and then (from c. 2000) decreasing occurrences (acidofrequent species), (4) species with widely increasing occurrences in recent decades (nitrofrequent species), and (5) species with rapidly increasing occurrences (rapidly spreading species of uncertain reasons). The discussed trends are known for some species at a global scale or European level, other examples are characteristic for Central Europe or Hungary. By studying the distribution maps of lichen bioindicators, tendencies of climate change and type of pollution can be determined and further changes can be predicted.

**Abstract:**

Distribution data originating from earlier herbarium collections and recent biodiversity records form the basis of distribution analyses in lichen species with different ecological requirements, where the records allowed comparisons or showed clear trends. As the occurrences of lichens are strongly correlated to background environmental conditions (e.g., air pollution, global warming), confirmed by Wirth’s ecological indicator values, the analysis of distribution types has a great value for bioindication and the establishment of current and future climatic and pollution situations. Five distribution types were introduced—presented by characteristic examples (13)—according to lichen distribution maps prepared in different periods of time (representing changing environmental conditions): (1) species of decreasing occurrences by time (e.g., *Lobaria pulmonaria*, *Menegazzia terebrata*, suboceanic, acidic pollution sensitive species), (2) species with no or few former records but with increasing occurrences in recent decades (e.g., *Flavoparmelia soredians*, *Hyperphyscia adglutinata*, *Solenopsora candicans*, sub-Mediterranean species), (3) species with increasing and then (from c. 2000) decreasing occurrences (e.g., *Scoliciosporum chlorococcum*, *Straminella conizaeoides*, acidofrequent species), (4) species with widely increasing occurrences in recent decades (e.g., *Physcia aipolioides*, *Piccolia ochrophora*, *Xanthoria parietina*, nitrofrequent species), and (5) species with rapidly increasing occurrences (e.g., *Absconditella lignicola*, *Coenogonium pineti*, *Evernia divaricata*, rapidly spreading species). The proposed distribution types of lichen species may be applied to wider regions (the European or the global level).

## 1. Introduction

Lichens are generally known as extremotolerant, cosmopolitan organisms found from the Equator to the poles and from sea level to the highest montane vegetation zones [1,2]. Even the simplest lichen consists of two different organisms [3]. Others (those of three to five partner symbioses) live together with additional fungal (parasitic or parasymbiont) or photosynthetic partners (green algae or cyanobacteria). Recently basidiomycete yeasts [4] and specific coexisting bacteria (most frequently Alphaproteobacteria) have also been discovered [5] to form a close connection with lichen association [6].

The indicator nature of lichens was recognized almost two centuries ago in relation to the environmental effects of Britain’s strong industrialization. It was observed [7] that the cover and the composition of lichen assemblages are related to air quality. Mapping of lichens revealed that the distribution of lichens according to their ecological requirements appears as variously polluted zones (central lichen desert, struggle zone, normal/natural zone) in cities and around factories and industrial plants [8,9,10]. The occurrence of particular species could be related to the concentration of SO_2_, being the most characteristic pollutant in the 1970s [11]. The study of lichens and air pollution soon became one of the leading directions of lichenological research; later investigations proceeded from the community and the species levels toward the cell and the molecular levels [12,13,14,15]. With the decline of SO_2_ pollution, it soon became obvious that lichens recolonize former lichen deserts [16,17], but the new assemblages consisted of species tolerating changed types of pollutants (e.g., NH_3_) [18,19,20,21,22].

Earlier the focus was on the composition and the distribution of species, later changes were due to time and environmental quality (corresponding to the quantitative and the qualitative nature of the pollution) [20,21,22,23,24,25].

Investigations concerning the indicator nature of lichens were extended to the monitoring of land use intensity, and they were studied in a wider context [26,27,28,29], with due attention also given to the effect of the substrate [30,31,32,33,34]. For monitoring studies, the time passed has a key importance [35]. Recognizing that global environmental changes mean an increasing pressure to the living organisms of the earth [36,37,38,39], it was shown that lichenized associations represent a sensitive model ecosystem in the changing environment [1,26,28]. Comparing distribution maps of various species in different periods of time and geographic scales is a tool that easily presents and illustrates background environmental changes [40,41,42]. The analysis of Hungarian data may result in similar examples [9,10,26,28].

The large amount of data accumulated during the past centuries makes a distribution analysis at a wide scale in time and in space possible. Altogether 55,000 lichen specimens are deposited in Hungarian herbaria collected between 1762 and 2022. Although these collections and additional literature records are not homogeneous in space and in time, the distribution maps of each species—prepared according to these records—are obviously applicable for recognizing characteristic distribution types in correlation with tendencies in the changing environment because of the indicator nature of lichens [7,8,9,10,11,12,13,14,15,16,17,18,19,20,21,22,23,24,25].

The main aims of this study are to prepare distribution maps of lichens in Hungary in various time periods and to introduce distribution types by comparing these maps and the direction of environmental changes. If possible, indicator groups of species are also going to be established on the basis of characteristic distribution types. We hypothesize that environmental changes are reflected in lichen distributions since lichens are regarded as sensitive environmental bioindicators. Though lichens as a group have a wide distribution, lichen species are characterized with specific and contrasting environmental requirements.

## 2. Materials and Methods

Hungary has a varied relative relief ranging from 0 m (at lowland) to *c*. 1000 m (at lower montane regions) with geomorphological origin–mostly marine, fluvial, and aeolian sediment of various ages and in smaller areas volcanic rocks covering the formations of older geographical periods. The six main geomorphological districts are the Alföld (the Great Hungarian Plain), the Kisalföld (the Little Hungarian Plain), the Alpokalja (the Foot of the Alps), the Transdanubian Hills, the Transdanubian Range, and the North Hungarian Range (as a part of the Northwestern Carpathians) [43,44]. The natural Pannonian vegetation consists of various deciduous forests (supplying a large number of tree species for epiphytic lichens) on mountainous regions and montane rocky and lowland sandy grasslands with special microclimatic conditions allowing lichen colonization on various soil and rock surfaces [45].

The macroclimate is predominantly continental with oceanic and Mediterranean effects. The annual sunshine duration is between 1900 and 2100 h increasing from northwest to southeast with an average monthly duration between 50 and 280 h (maximum in June, minimum in January [46]. The difference in monthly mean temperature is more than 1 °C if the current (1981–2010) 30-year period is compared to 1960–1990 (which is often considered as a reference period in modelling future climate) [46]. The mean temperature is currently (1981–2010) 10.35 °C. It has increased by 1.6 °C (reaching even 2 °C in summer) in this 30-year period. According to predicting models, the mean temperature will continue to increase both annually and seasonally by 1–2 °C for 2021 to 2050 and by 3–4 °C for 2071–2100 and from west-northwest to east-southeast. The highest increase 3.5–4.5 °C is expected in summer (even by 6 °C in August) [46]. The frequency of days with frost will be significantly lower than in the past. Annual precipitation is now 580 mm (1981–2010), ranging from 700–800 mm (in mountains and at the southwestern border) to 500 mm in the Alföld. It is predicted to decrease slightly (by c. 15–20 mm) for 2021–2050, particularly in July–August and January–February. However, there is a precipitation increase in autumn. Furthermore, different predictions exist for the period 2071–2100, showing either a further decrease or an increase in precipitation.

The atmospheric concentration of sulphur dioxide, creating the major part of acidic deposition, increased until the middle of the 1980s (reaching 25–30 µg/m^3^ SO_2_) in Hungary, after that it has been continuously decreasing (reaching 1–3 µg/m^3^ SO_2_ in 2015) [47]. The household emission of nitrogen-containing pollutants—with a significant effect on lichen colonization in and around settlements—increased considerably (from 1955 t NH_3_ in 1990 to 5672 t NH_3_ in 2017) [48].

A detailed study on lichen-forming fungi of Hungary was published by Klára Verseghy in 1994 [49]; however, this identification key contained distribution data of lichens by geographic regions only, still it was very useful since it collected data scattered in a large number of literature sources. In 2009, a revised checklist of species [50] recorded from Hungary was compiled from literature and herbarium records (from the late 1800s to 2022). The checklist was continuously updated, and the species number has now reached 926 due to our revision and mainly our own collections from 1979. Most of the species (49%) are saxicolous on various rocks, 15% are terricolous, 33% are corticolous, and 3% are lignicolous. These species and their records (c. 55,000) form the basis of our studies. The number of references checked is c. 180 from 1869 to 2021. New collections were identified with the aid of various literature sources mainly [51,52]. The morphology and the anatomy were studied by using a NIKON Eclipse/NiU (DIC, epifluorescence) compound microscope, Nikon SMZ18 stereo microscope as well as Olympus SZX9 and Olympus BX50 (DIC) microscopes. Micrographs were prepared by Olympus E450 camera (with Quick Photo Camera 2.3 software) and Nikon DS-Fi1c and Fi3 camera (with NIS-Elements BR ML software), with the indicated microscopes. HPTLC analysis was carried out according to standard methods for analyzing lichen samples [53,54], where it was necessary. The nomenclature mainly follows the IndexFungorum [55].

Voucher specimens are deposited in herbaria BP, BTM, DE, EGR, GODO, JPU, SZE, SAMU, and VBI (abbreviations mainly follow [56]). Most of the specimens studied are in BP (c. 40,000 specimens), and a c. 15,000 specimens are found in other herbaria.

Distribution maps were constructed by a computer program for geographical information systems, QGIS 3.18.2 ‘Zürich’, released in 2020, applying an adaptation of the Central European grid system [57,58]. The illustrated symbols (dots) represent units of *c*. 5 km × 6 km. In 1989, a European Lichen Mapping project was initiated [59,60,61]. Data before and after 1975 were distinguished, considering changes in conditions of air pollution in Europe. Therefore, these periods are shown on maps, although the decline of acidic pollution started 10–15 years later in Hungary (cf., [46]).

## 3. Results

Distribution maps of a wide selection of species from the Hungarian lichen flora [49,50] were prepared in recent decades. Former versions of some of these maps have been published in scattered publications on various taxa, e.g., [62,63]. Species with the best-known distribution records were selected to be presented here, where data from different time periods allowed reliable comparisons or recent data showed clear trends. The following distribution types are proposed for species on the basis of an analysis of historical and recent data:

(a) species with decreasing occurrences;
(b) species with no or few former records but with increasing occurrences in recent decades;
(c) species with increasing, then decreasing occurrences;
(d) species with widely increasing occurrences in recent decades;
(e) species with rapidly increasing occurrences.

In order to evaluate all of the 926 species into these distribution types, a few examples were analyzed in more detail. Nevertheless, a considerable number (c. 40%) were categorized as “distribution types to be established” due to the lack of sufficient data or having only uncertain records.

**Figure 1:**
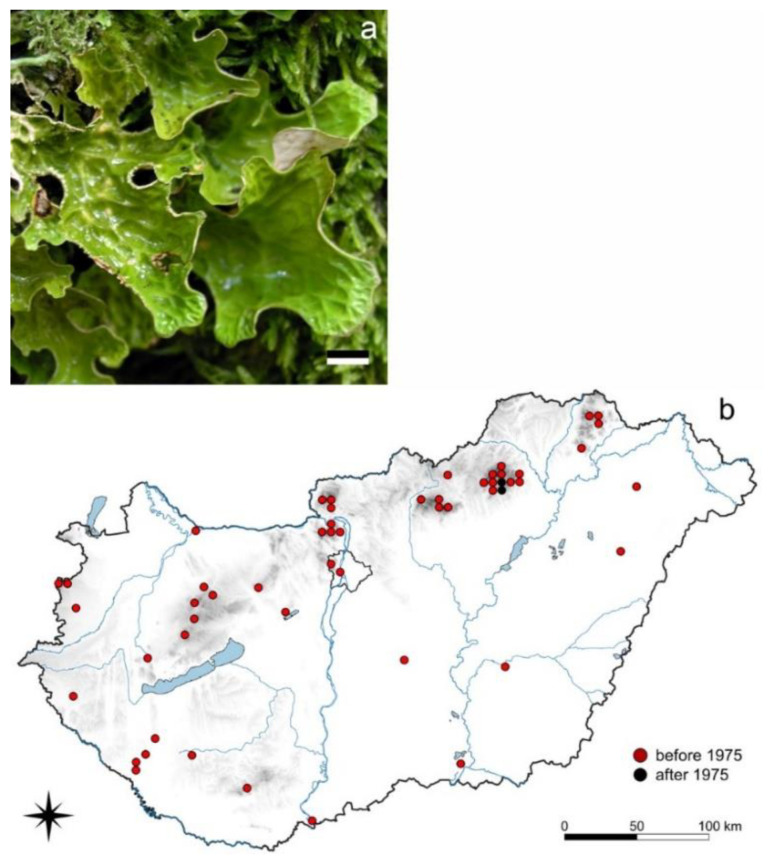
*Lobaria pulmonaria* (**a**) habit (scale 1 cm); (**b**) its distribution in Hungary (94 records). Dots represent c. 5 km × 6 km areas. (Photo: L. Lőkös).

**Figure 2:**
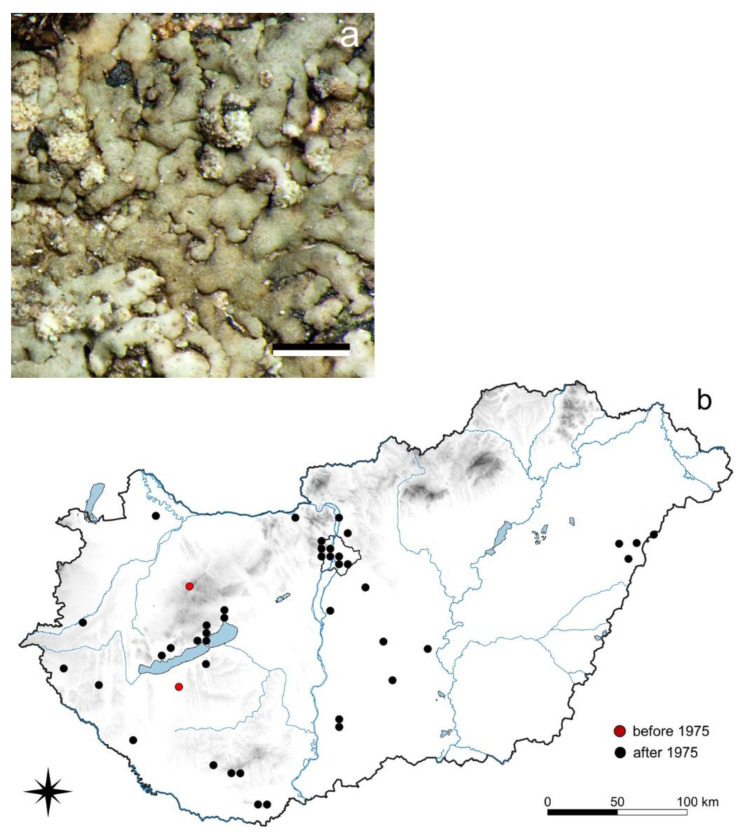
*Hyperphyscia adglutinata* (**a**) habit (scale 0.5 cm); (**b**) its distribution in Hungary (56 records). Dots represent c. 5 km × 6 km areas. (Photo: L. Lőkös).

**Figure 3:**
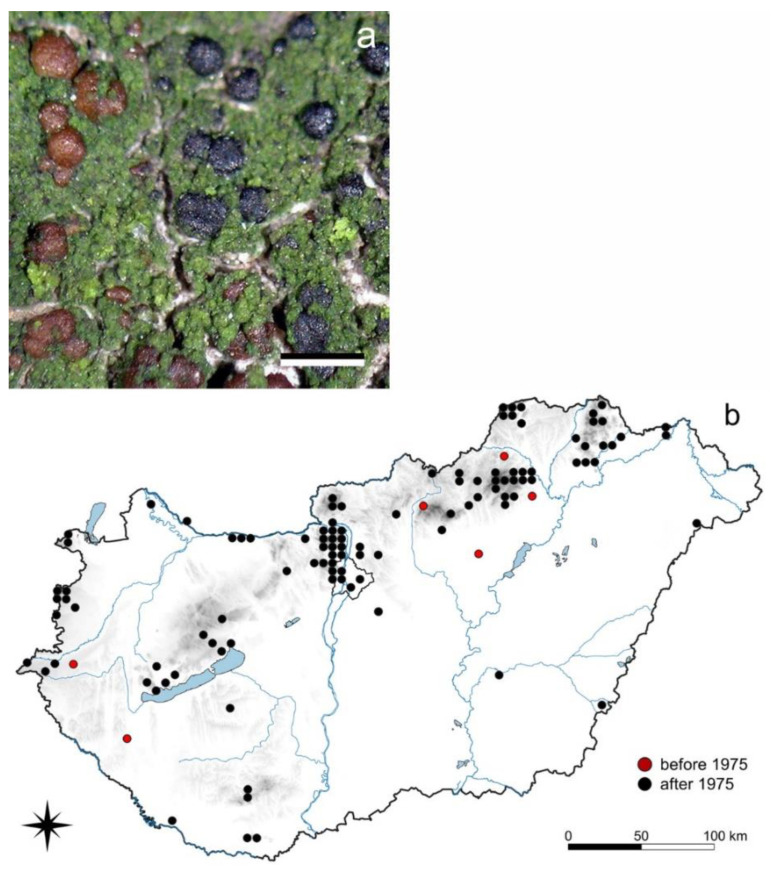
*Scoliciosporum chlorococcum* (**a**) habit (scale 0.5 mm); (**b**) its distribution in Hungary (322 records altogether). Dots represent c. 5 km × 6 km areas. (Photo: E. Farkas).

**Figure 4:**
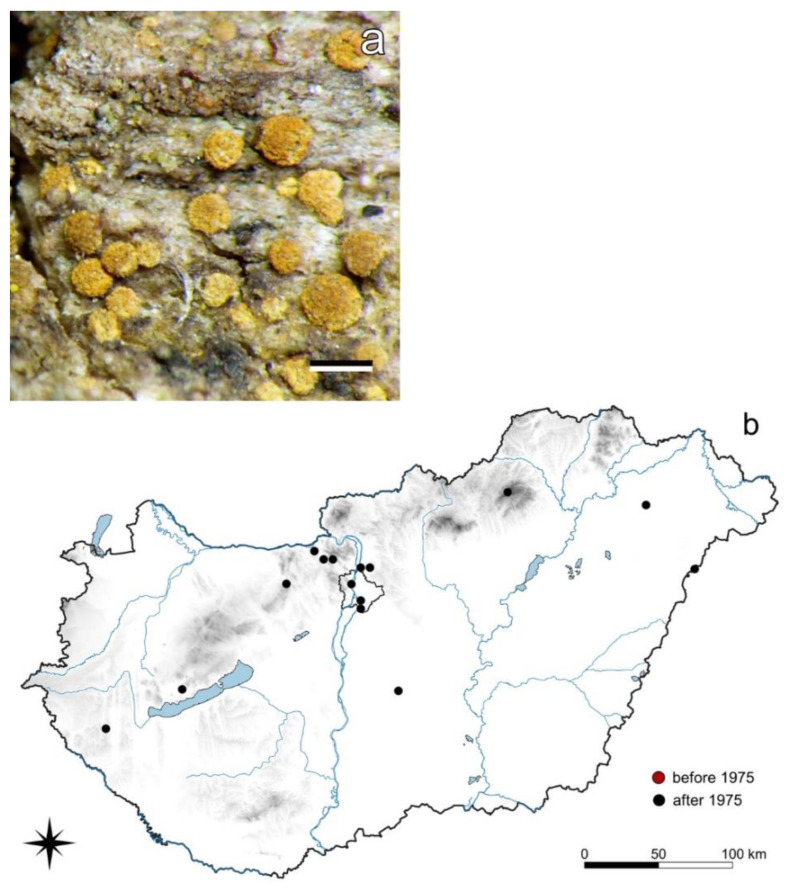
*Piccolia ochrophora* (**a**) habit (scale 0.5 mm); (**b**) its distribution in Hungary (16 records). Dots represent c. 5 km × 6 km areas. (Photo: E. Farkas).

**Figure 5:**
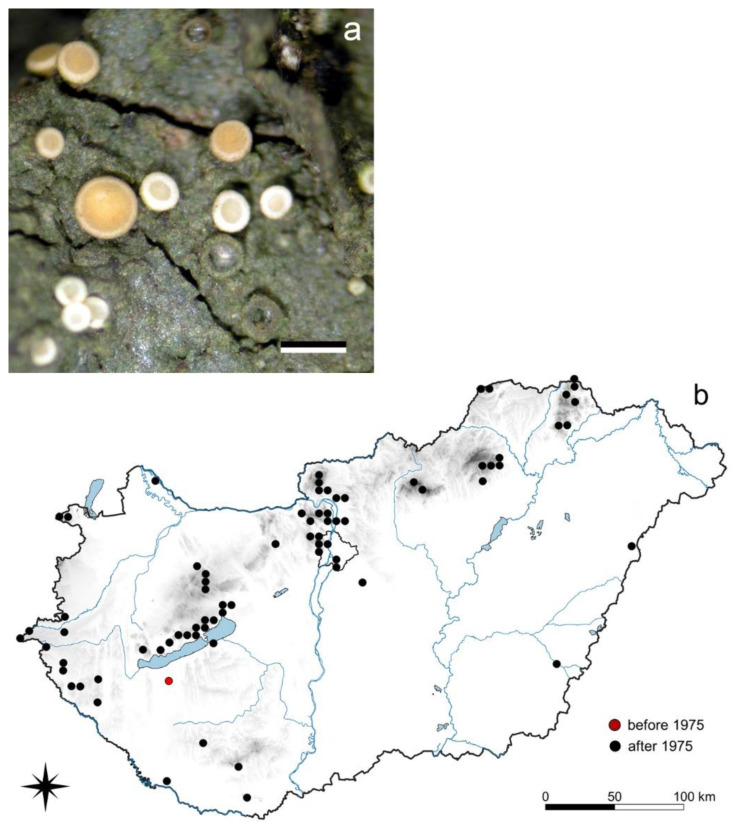
*Coenogonium pineti* (**a**) habit (scale 0.5 mm); (**b**) its distribution in Hungary (101 records, 1954–2022). Dots represent c. 5 km × 6 km areas. (Photo: E. Farkas).

Thirteen examples of the distribution types are presented and illustrated below ( Figure 1, Figure 2, Figure 3, Figure 4 and Figure 5; Figures S1–S8 in Supplementary File) and the possible reasons for these characteristic distributions are discussed later in Section 4.

### 3.1. Species of Decreasing Occurrences

#### 3.1.1. *Lobaria pulmonaria* L.

*Lobaria pulmonaria* is a very conspicuous, large, foliose species (Figure 1) [63]. In the past, it was widely distributed in Hungary in the mountain ranges (mainly the Bükk and Mátra Mts) occurring dominantly on bark of *Fagus sylvatica*, and to a lesser extent on other broad-leaved tree species (*Acer*, *Betula*, *Quercus*, *Ulmus* spp.) as well as on mossy andesitic rock surfaces. In older times, it was considered as a medicinal plant (as drug *Herba pulmonariae arboreae*, *Lichen pulmonarius*, or *Muscus pulmonarius*), and it was used against lung diseases, but no information is available regarding how extensively it was collected in Hungary for this purpose [64].

Altogether, 94 data were recorded from herbaria (BP, EGR, GODO, PECS, SZE, SZO) and literature up to 1967, but only three recent occurrences were found in the Bükk Mts in 2008 and 2016 [64].

#### 3.1.2. *Menegazzia terebrata* (Hoffm.) A. Massal.

*M. terebrata* is a characteristic foliose species having large, round perforations on its upper surface (Figure S1). Until 1960 only nine old records were known from suboceanic habitats in the mountain range of Hungary (e.g., Bükk, Mátra, Sopron, and Zemplén Mts), and it was recently confirmed at the former, single locality in the Zemplén Mts. It grows on bark (*Fagus*) and on mossy siliceous rocks in Hungary.

### 3.2. Species with No or Few Former Records but with Increasing Occurrences in Recent Decades

#### 3.2.1. *Flavoparmelia soredians* (Nyl.) Hale

*F. soredians* is originally an Atlanto-Mediterranean foliose species (Figure S2), which seems to be spreading in Hungary [65]. Its 19 Hungarian localities were discovered between 2011 and 2022. Contrary to its earlier known habitat requirements (sub-Mediterranean), it was collected mostly in anthropogenic, urban habitats. *F. soredians* is corticolous on different primarily eutrophicated phorophytes in Hungary, e.g., *Acer* sp., *Ailanthus altissima*, *Prunus* sp., *Quercus cerris*, *Q. petraea*, *Robinia pseudoacacia*, *Tilia* sp., and decaying wood.

#### 3.2.2. *Hyperphyscia adglutinata* (Flörke) H. Mayrhofer et Poelt

*H. adglutinata* is a small foliose species, with narrow (0.3–0.7(–2) mm wide) lobes and maculiform or capitate soralia (Figure 2). In addition to its four old occurrences from 1925 to 1960, it has been reported from several localities recently from 2000 to 2022, including urban habitats (e.g., [66,67,68]). It is a corticolous species growing on various tree species, frequently on eutrophicated bark (*Acer campestre*, *A. pseudoplatanus*, *A. tataricum*, *Alnus glutinosa*, *Betula pendula*, *Cornus mas*, *Corylus avellana*, *Cotinus coggygria*, *Fraxinus ornus*, *Morus alba*, *Populus canescens*, *Prunus cerasus*, *Pyrus pyraster*, *Quercus pubescens*, *Robinia pseudoacacia*, and *Sambucus nigra*).

#### 3.2.3. *Solenopsora candicans* (Dicks.) J. Steiner

*S. candicans* has a pale grey, thickly white-pruinose, placodioid, rosette-like thallus and dark brown to black apothecia (Figure S3). Two old localities were known from the Buda Mts (1934) and the Balaton Upland (1947). Between 2007 and 2015, it was found at several localities in the Balaton Upland and the Buda Mts predominantly on calcareous rocks and also on geyserite near Tihany [69].

### 3.3. Species with Increasing, then Decreasing Occurrences

#### 3.3.1. *Scoliciosporum chlorococcum* (Stenh.) Vězda

*S. chlorococcum* is a corticolous crustose species with a green granulose thallus and shiny blackish or reddish-brown apothecia of 0.2–0.3 mm diam. (Figure 3). Only 15 records are known between 1912 and 1974, while 292 records were made between 1976 until 2004 [70], but only 15 records between 2005 and 2016. In Hungary it was collected predominantly from phorophytes of acidic bark (*Fagus sylvatica*, *Juniperus communis*, *Larix decidua*, *Picea abies*, *Pinus nigra*, *P. sylvestris*, and *Quercus cerris*) and a great number of other species (*Acer campestre*, *A. platanoides*, *A. pseudoplatanus*, *Aesculus hippocastanum*, *Alnus glutinosa*, *Armeniaca vulgaris*, *Betula pendula*, *B. pubescens*, *Carpinus betulus*, *Castanea sativa*, *Cerasus avium*, *Cotinus coggygria*, *Crataegus monogyna*, *Euonymus verrucosus*, *Fraxinus excelsior*, *F. ornus*, *Juglans regia*, *Malus domestica*, *Morus alba*, *Persica vulgaris*, *Populus alba*, *P. tremula*, *Prunus domestica*, *Pyrus achras*, *P. communis*, *Quercus farnetto*, *Q. petraea*, *Q. pubescens*, *Q. robur*, *Robinia pseudoacacia*, *Salix* sp., *Sorbus* sp., *S. torminalis* and *Tilia* spp.) as well as from decaying wood.

#### 3.3.2. *Straminella conizaeoides* (Cromb.) S. Y. Kondr., Lőkös et Farkas

*S. conizaeoides* is a corticolous crustose species with a greyish-green granular often sorediate thallus containing fumarprotocetraric acid. The apothecia are frequent, discs are pale green-grey to grey-brown, with a thalline margin (Figure S4). Only 46 records were known between 1920 and 1961, with 191 records between 1978 until 2004, and only 4 between 2005 and 2021. In Hungary it has been collected mainly from phorophytes of acidic bark (*Fagus sylvatica*, *Larix decidua*, *Pinus nigra*, *P. sylvestris*, and *Quercus cerris*) and a great number of other species (*Acer campestre*, *A. platanoides*, *A. pseudoplatanus*, *Aesculus hippocastanum*, *Alnus glutinosa*, *Armeniaca vulgaris*, *Betula pendula*, *Carpinus betulus*, *Castanea sativa*, *Cerasus avium*, *Crataegus monogyna*, *Euonymus verrucosus*, *Fraxinus excelsior*, *F. ornus*, *Juglans regia*, *Malus domestica*, *Persica vulgaris*, *Populus* sp., *Pyrus achras*, *Quercus petraea*, *Robinia pseudoacacia*, *Sorbus torminalis*, *Tilia argentea*, and *T. platyphyllos*) as well as from decaying wood. The species grows frequently together with *Hypogymnia physodes* and *Scoliciosporum chlorococcum*.

### 3.4. Species with Widely Increasing Occurrences

#### 3.4.1. *Physcia aipolioides* (Nádv.) Breuss et Türk

*P. aipolioides* is a nitrofrequent species with a relatively large and thick thallus and slightly pruinose lobes [71] (Figure S5). Formerly, it was collected from the Sopron Mts and in the Balaton region, and recently from several new localities near settlements, including several localities also in Budapest. It is corticolous on various tree species (*Acer platanoides*, *A. pseudoplatanus*, *Aesculus hippocastanum*, *Fraxinus* sp., *Gleditsia triacanthos*, *Juglans regia*, *Populus × euramericana*, *P. nigra*, *Quercus pubescens*, *Q. robur*, *Robinia pseudoacacia*, and *Ulmus campestre*).

#### 3.4.2. *Piccolia ochrophora* (Nyl.) Hafellner

*P. ochrophora* is a corticolous crustose lichen species with convex brownish or ochraceous orange apothecia covered by thick orange pruina. It has only been collected from 1987 to 2018 [72]. Being moderately nitrofrequent [52], it grows under ruderal conditions on eutrophicated bark of *Acer campestre*, *Populus canescens*, *Populus* spp., *Robinia pseudoacacia*, *Salix alba* or *Sambucus nigra* (Figure 4).

#### 3.4.3. *Xanthoria parietina* (L.) Th. Fr.

*X. parietina* is a widespread, nitrofrequent, conspicuous, foliose species with yellow to orange thallus and large lecanorine apothecia, with an orange disc (Figure S6). It was also widely distributed throughout Hungary in older times (more than half of the records are between 1870 and 1975). Later it was also observed in several localities, even in the former lichen deserts of big cities. It mainly colonizes tree bark, but it is often found on wood, rocks, and concrete. It grows on a great number of phorophytes (mostly on eutrophicated bark), e.g., *Acer campestre*, *A. negundo*, *A. platanoides*, *A. pseudoplatanus*, *Aesculus hippocastanum*, *Ailanthus altissima*, *Alnus* sp., *Amygdalus communis*, *Armeniaca vulgaris*, *Artemisia monogyna*, *Berberis vulgaris*, *Betula pendula*, *Carpinus betulus*, *Castanea sativa*, *Celtis occidentalis*, *Cerasus avium*, *Cotinus coggygria*, *Crataegus monogyna*, *Elaeagnus angustifolia*, *Euonymus* sp., *Fagus sylvatica*, *Fraxinus excelsior*, *F. ornus*, *Fumana procumbens*, *Gleditsia triacanthos*, *Juglans nigra*, *J. regia*, *Juniperus communis*, *Kochia prostrata*, *Koelreuteria paniculata*, *Maclura pomifera*, *Malus pumila*, *Morus alba*, *M. nigra*, *Picea abies*, *Pinus nigra*, *P. sylvestris*, *Populus alba*, *P. canadensis*, *P. × euramericana*, *P. italica*, *P. nigra*, *P. pyramidalis*, *P. tremula*, *Prunus armeniaca*, *P. cerasus*, *P. domestica*, *P. mahaleb*, *P. spinosa*, *Pyrus achras*, *P. pyraster*, *Quercus cerris*, *Q. pubescens*, *Q. robur*, *Q. rubra*, *Ribes grossularia*, *Robinia pseudoacacia*, *Salix alba*, *S. fragilis*, *Sambucus nigra*, *Sorbus torminalis*, *Syringa vulgaris*, *Tamarix gallica*, *Taxodium distichum*, *Thuja occidentalis*, *Tilia* sp., *Ulmus campestris*, and *U. glabra*.

### 3.5. Species with Rapidly Increasing Occurrences

#### 3.5.1. *Absconditella lignicola* Vězda et Pišút

*A. lignicola* is a crustose, primarily lignicolous species (Figure S7). Its thin thallus is vivid or dark greenish-brownish, or inconspicuous, usually growing together with a gelatinous algal biocrust. The apothecia are tiny (0.1–0.3 mm), pale waxy cream or ivory whitish, scattered, sessile, and usually concave, urn-like [73].

Hungarian specimens were collected from decaying wood of *Picea abies*, *Pinus nigra* and *P. sylvestris* in both montane and lowland habitats, mostly in lowland pine plantations, but also in seminatural forests, accompanied by *Micarea denigrata*, *Placynthiella icmalea*, and *Trapeliopsis flexuosa*.

#### 3.5.2. *Coenogonium pineti* (Ach.) Lücking et Lumbsch

*C. pineti* is a corticolous crustose species with tiny whitish, pale orange apothecia of 0.2–0.4 mm diameter [74] (Figure 5). Prior to 1975, only two old records were known (1954 Buzsák and 1974 Mátra Mts, Ágasvár); since then, a further 99 records have been added. Thalli of *C. pineti* most frequently colonize the base of *Alnus glutinosa* and *Sorbus torminalis* tree trunks, but it is also found on *Acer campestre*, *A. platanoides*, *Carpinus betulus*, *Cornus mas*, *Fagus sylvatica*, *Fraxinus excelsior*, *Larix decidua*, *Picea abies*, *Pinus nigra*, *P. sylvestris*, *Pseudotsuga* sp., *Pyrus pyraster*, *Quercus cerris*, *Q. petraea*, *Q. pubescens*, and *Salix* sp.; they even occur on *Robinia pseudoacacia*, where the trunk can be covered up to 2 m.

#### 3.5.3. *Evernia divaricata* (L.) Ach.

*E. divaricata* is a fruticose species with greyish-green to yellowish-green flattened lobes, and short, thorn-like, divaricate side branches (Figure S8). Four records were known between 1866 and 1932, and 31 records between 2000 and 2022, sometimes spreading into unusual habitats. In natural habitats its thalli are pendent, beard-like, however Hungarian specimens are mostly, compact, shrub-like. It is mostly corticolous growing on various tree species (*Acer platanoides*, *Ailanthus altissima*, *Betula pendula*, *Crataegus monogyna*, *Picea abies*, *Populus alba*, *P. nigra*, *Prunus spinosa*, *Quercus cerris*, *Q. petraea*, *Sorbus domestica*), but lignicolous specimens are also known.

## 4. Discussion

**Figure 6:**
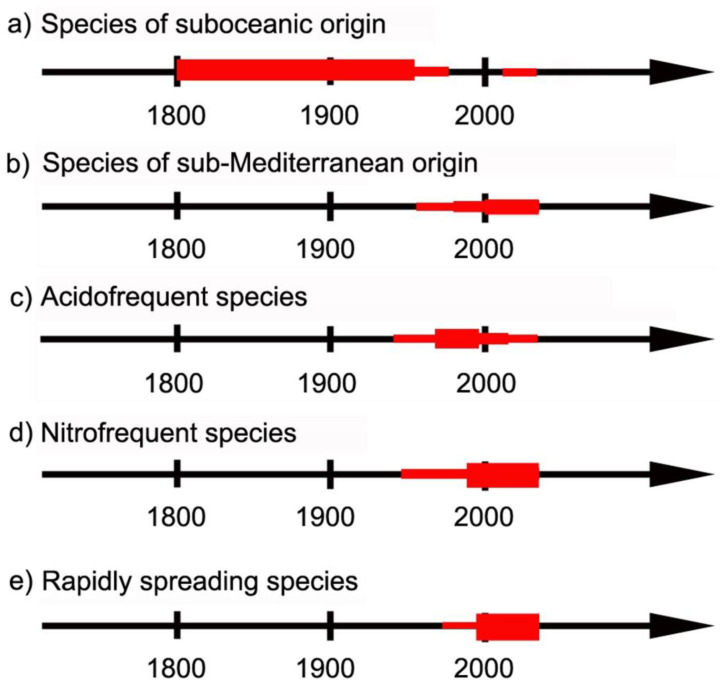
The five changing distribution types of lichen species in time (years) according to possible explanations represented by the thickness of the red line.

In comparing the various distribution maps based on historical and recent records by studying (1) the direction of changes, (2) the possible environmental changes, and (3) the ecological indicator values by Wirth [75], the following explanation for the various groups can be suggested (Figure 6).

### 4.1. Species of Suboceanic Origin

Species of decreasing occurrences (Section 3.1) belonging to this group (e.g., *Lobaria pulmonaria*, *Menegazzia terebrata*—Figure 1 and Figure S1) were considerably or moderately frequent before the period with strong acidic air pollution (caused by SO_2_ pollution from traditional heating in households, from industry and from coal heated power stations). Their natural distribution is also characteristic of more humid climatic conditions than those currently occurring in Hungary. They are more frequent at higher elevation and among suboceanic conditions.

Though the decreasing SO_2_ pollution level (under 30 µg/m^3^ SO_2_ according to [76]) could result in the re-colonization of *Lobaria pulmonaria*–as recorded in 2009 [63], the drier and warmer climate is not supporting the return of this species in larger abundance in the country. Because of the general decline of its populations worldwide, various aspects of its dispersal (cf. also land use history) and population dynamics were studied [77,78,79,80,81,82,83,84]. It corresponds with ecological indicator values by Wirth [75], especially those of acidity (4–5) and humidity (7). Its decline from its former habitats was most probably also due to the decrease in natural forest ecosystems [48], which resulted in changing climatic, especially microclimatic conditions (e.g., air humidity) important for this species. According to Rose [85], *Lobaria amplissima*, *L. pulmonaria*, and *L. scrobiculata* are regarded as indicators of ecological continuity. *Lobaria pulmonaria* was found to be a key species in primeval forest preservation as an “old forest indicator” in the Eastern Carpathians [86].

Several other oceanic/suboceanic and alpine species show a declining tendency in Hungary probably due to changes to a drier and a warmer climate; these are: e.g., *Bacidia rosella*, *Cetrelia* spp., *Cladonia portentosa*, *Fuscopannaria leucophaea*, *Heterodermia speciosa*, *Leptogium cyanescens*, *Normandina pulchella*, *Parmeliella triptophylla*, *Parmotrema crinitum*, *P. perlatum*, *Peltigera collina*, *Pertusaria hemisphaerica*, and *Umbilicaria cylindrica*.

Some of the species related to this type have a slightly increasing distribution, e.g., *Ochrolechia arborea* [87], *Peltigera leucophlebia* [62], and the recently found *Xanthoparmelia mougeotii* [88].

### 4.2. Species of Sub-Mediterranean Requirements

Most of these lichens, with no or few former records but with increasing occurrences in recent decades (Section 3.2), were not collected in the 20th century and several species only appeared in Hungary during the past 10–20 years (e.g., *Flavoparmelia soredians*—Figure S2 [65]; *Hyperphyscia adglutinata*—Figure 2; *Solenopsora candicans*—Figure S3 [69]).

Since these lichens are still rarely collected in Hungary, it is difficult to explain their presence. Some hypotheses, such as that described by Wirth in 1997 [40] may provide some explanation since some lichens may expand their distribution. The background to these changes may be a joint effect of an as yet unknown cause, such as a minor environmental change to the microhabitat of the species and changed characters (morphological, physiological, and/or chemical) due to a possible change in the expression of genes [89,90,91,92]. *Flavoparmelia soredians* was also collected in urbanized places, outside of its preferred suboceanic habitats. Wirth [40] also suggested the decrease in the SO_2_ level and the change of climate to a milder one in Germany as a reason. Seaward and Coppins [93] mention hypertrophication as the reason for its spread in the British Isles. Anthropogenic effects are also confirmed by Nygaard and Tønsberg [94] for its immigrant nature in Norway.

*Solenopsora candicans* was overlooked in Hungary but collected recently at several new localities [69]. The recent spread of *Hyperphyscia adglutinata* is even more obvious (Figure 2); it is regarded as a sub-Mediterranean element, growing on nutrient rich trees frequently in well illuminated conditions [95].

All these species are of sub-Mediterranean origin, but due to climatic warming they have found more advantageous conditions and therefore are spreading in Hungary. Further species with a similar distribution type in Hungary are *Leptogium ferax* [96], *Parmelia submontana* Hale [97], and *Xanthoparmelia verrucigera*. Among the ecological indicator values by Wirth [75], temperature (value 9) mostly justifies the change in distribution for these species.

### 4.3. Acidofrequent Species

*Scoliciosporum chlorococcum*, *Straminella conizaeoides*, *Hypogymnia physodes*, and *Lepraria incana* are more difficult to find recently than a few decades ago. These are species with increasing, then decreasing occurrences (Section 3.3.); especially, *Scoliciosporum chlorococcum* (Figure 3) and *Straminella conizaeoides* (Figure S4) with hardly any herbarium records since the beginning of the 20th century in Hungary, while they were reported in lists indicating acidic air pollution [11] and later also discovered in Budapest, and these were among its most frequent species [10,98]. Since acidic air pollution has decreased considerably [46], these acidofrequent species, tolerating acidic air pollution, have been disappearing since 2000. Both species have ecological indicator values of 2–3 by Wirth [75], which are characteristic of an acid environment.

### 4.4. Nitrofrequent Species

While acidic air pollution is decreasing, nitrogen-containing pollutants (NO_x_ and NH_3_) are increasing, especially in and near settlements [46,47,93]. These conditions have affected the distribution of the so-called nitrophilous species (e.g., *Phaeophyscia orbicularis*, *Xanthoria parietina*–Figure S6), however, we prefer a more neutral term: nitrofrequent. Their ecological indicator value of 8 by Wirth [74] in this respect is high. These species were already more or less frequent earlier, almost irrelevant of the level of acidic pollution, but when it started to decrease and nitrogen-containing pollutants increased (cf. Section 3.4), the nitrofrequent species spread and their distribution increased significantly. *Physcia aipolioides* (Figure S5) is an interesting example; in Slovakia, Czechia, and Austria, it demonstrates a similar continuous distribution type with a few outliers in fairly remote localities. An increase in the number of localities in Czechia and Slovakia, together with the toxitolerance of the lichen, led in the 1980s and the 1990s to the assumption that the species spread invasively [71]. The lichen also occurs in Hungary, and the records from Bulgaria and Montenegro indicate that its distribution area is probably even much larger. The lichen has the potential to spread further.

The species increasing under nitrogen-rich conditions in Hungary are *Amandinea punctata*, *Candelariella reflexa*, *Catillaria nigroclavata*, *Lecania cyrtella*, *L. naegelii*, *Lepraria elobata*, *L. lobificans*, *Phaeophyscia orbicularis*, *Physcia adscendens*, *P. aipolioides* (Figure S5), *Piccolia ochrophora* (Figure 4), and *Xanthoria parietina* (Figure S6).

### 4.5. Rapidly Spreading Species of Uncertain Reason

The distribution tendencies of certain species, namely the rapid and sudden increase of occurrences (cf. Section 3.5), makes them very similar to invasive vascular plant species [99]. The earliest Hungarian specimens of *Coenogonium pineti* were collected in Buzsák (Belső-Somogy) in 1954, almost 20 years later in valley “Csörgő-patak völgye,” Mátra Mts [100], then in Barcsi Ősborókás TvT and in Zselic in 1987 [101]. From 1991, several new localities were recognized practically from all regions of the country. These collection data suggest that changes of environmental conditions in recent decades support the distribution of this species throughout Hungary, but no closer explanation was found for its spread.

*Evernia divaricata* also had scattered records in the past, but in recent decades it is collected more and more frequently in Hungary. Another example is *Absconditella lignicola* [73]. It was found in 2009 in the Őrség (W Hungary), then it was collected from 34 localities in the Börzsöny, Buda, and Mátra Mts, the Danube–Tisza Interfluve, the Transdanubian Hills, the Nyírség, and the Őrség.

It is difficult to explain the status of the above species, and most probably a combination of several reasons (e.g., pollution, preference of climate or substrate) are responsible for their appearance and spread. The various ecological indicator values (by Wirth [75]) themselves do not explain the distributions since the different values characterizing the species fall in a wide range or they show even opposite trends.

## 5. Conclusions

The study of a large amount of herbarium material and literature records made it possible to establish different distribution types of lichens. The analyses—also based on literature sources of background data—including Wirth’s ecological indicator values [75], have justified the possible reasons for the appearance of these distribution types.

The discussed trends are known for some species at a global scale or European level, other examples are characteristic for Central Europe or Hungary. Since the distribution is strongly correlated to background environmental conditions (e.g., air pollution, land use intensity, global warming, substrate type), the analyses of distribution maps have a great value in biomonitoring. By studying the distribution maps of lichen bioindicators, tendencies of climate change and type of pollution can be determined and further changes can be predicted. However, further extended collections with precise collection design in space and time are necessary to provide current distributional data suitable for a statistical analysis.

## Data Availability

The following public databases were used: IndexFungorum (http://www.indexfungorum.org/, accessed on 27 September 2021); Index Herbariorum (http://sweetgum.nybg.org/science/ih/, accessed on 27 September 2021); MycoBank (https://www.mycobank.org/, accessed on 27 September 2021); Recent literature on lichens (http://nhm2.uio.no/botanisk/lav/RLL/RLL.HTM, accessed on 27 September 2021). The data presented in this study are available in the referred publications [49,63,65,69,70,71,72], if otherwise not, then these are available on request from the corresponding author [E.F.]. Further records (herbarium and literature) are summarized in Table S1 [link for supplement of current paper is to be added here]. Data of herbarium records are available upon request from the curators of the given herbaria [see Index Herbarium at above link].

## Supplementary Materials

The following supporting information can be downloaded at: https://www.mdpi.com/article/10.3390/jof8060600/s1, Figure S1: *Menegazzia terebrata* (a) habit (scale 1 cm); (b) its distribution in Hungary (10 records). Dots represent c. 5 × 6 km areas. (Photo: © E. Timdal); Figure S2: *Flavoparmelia soredians* (a) habit (scale 0.5 cm); (b) its distribution in Hungary (19 records). Dots represent c. 5 km × 6 km areas. (Photo: E. Farkas); Figure S3: *Solenopsora candicans* (a) habit (scale 0.5 cm); (b) its distribution in Hungary (13 records). Dots represent c. 5 km × 6 km areas. (Photo: E. Farkas); Figure S4: *Straminella conizaeoides* (a) habit (scale 0.1 cm); (b) its distribution in Hungary (241 records altogether). Dots represent c. 5 km × 6 km areas. (Photo: E. Farkas); Figure S5: *Physcia aipolioides* (a) habit (scale 0.5 cm); (b) its distribution in Hungary (31 records). Dots represent c. 5 km × 6 km areas. (Photo: L. Lőkös); Figure S6: *Xanthoria parietina* (a) habit (scale 1 cm); (b) its distribution in Hungary (1023 records). Dots represent c. 5 km × 6 km areas. (Photo: E. Farkas); Figure S7: *Absconditella lignicola* (a) habit (scale 0.5 cm); (b) its distribution in Hungary (37 records, 2009–2022). Dots represent c. 5 km × 6 km areas. (Photo: E. Farkas); Figure S8: *Evernia divaricata* (a) habit (scale 1 cm); (b) its distribution in Hungary (35 records). Dots represent c. 5 km × 6 km areas. (Photo: G. Matus); Table S1: Distribution records of illustrated species with main literature sources (containing published distribution records) and additional herbarium records (herbarium code, locality code [57,58], year).
