# Supplementary material for: Distribution Types of Lichens in Hungary That Indicate Changing Environmental Conditions
Source: J Fungi (Basel). 2022 Jun 3;8(6):600. doi: 10.3390/jof8060600 (PMC9225213; doi:10.3390/jof8060600)
Supplement: Supplementary file 1 [file jof-08-00600-s001.zip › Supplement for Article jof-1722175_ 29052022.pdf]

Supplement for Article

Distribution types of lichens in Hungary that indicate changing environmental conditions

Edit Farkas [1,*], Nóra Varga [1], Katalin Veres [1], Gábor Matus [2], Mónika Sinigla [3] and László Lőkös [4]

[1] Institute of Ecology and Botany, Centre for Ecological Research, H–2163 Vácrátót, Hungary; farkas.edit@ecolres.hu; varga.nora@ecolres.hu; veres.katalin@ecolres.hu
[2] Department of Botany, Faculty of Science and Technology, University of Debrecen, H–4010 Debrecen, Hungary; matus.gabor@science.unideb.hu
[3] Bakony Museum of the Hungarian Natural History Museum, H–8420 Zirc, Hungary; monikasinigla@gmail.com
[4] Department of Botany, Hungarian Natural History Museum, H–1431 Budapest, Hungary; lokos.laszlo@nhmus.hu
* Correspondence: farkas.edit@ecolres.hu

Supplementary Figures S1–S8
Supplementary Table S1

Citation: Edit Farkas [1,*], Nóra Varga [1], Katalin Veres [1], Gábor Matus [2], Mónika Sinigla [3] and László Lőkös [4] Distribution types of lichens in Hungary indicate changing environmental conditions. *J. Fungi* **2022**, *8*, 600. ttps://doi.org/10.3390/jof8060600

Academic Editor: Firstname Lastname

Received: date
Accepted: date
Published: date

Publisher's Note: MDPI stays neutral with regard to jurisdictional claims in published maps and institutional affiliations.

Copyright: © 2022 by the authors. Submitted for possible open access publication under the terms and conditions of the Creative Commons Attribution (CC BY) license (https://creativecommons.org/licenses/by/4.0/).

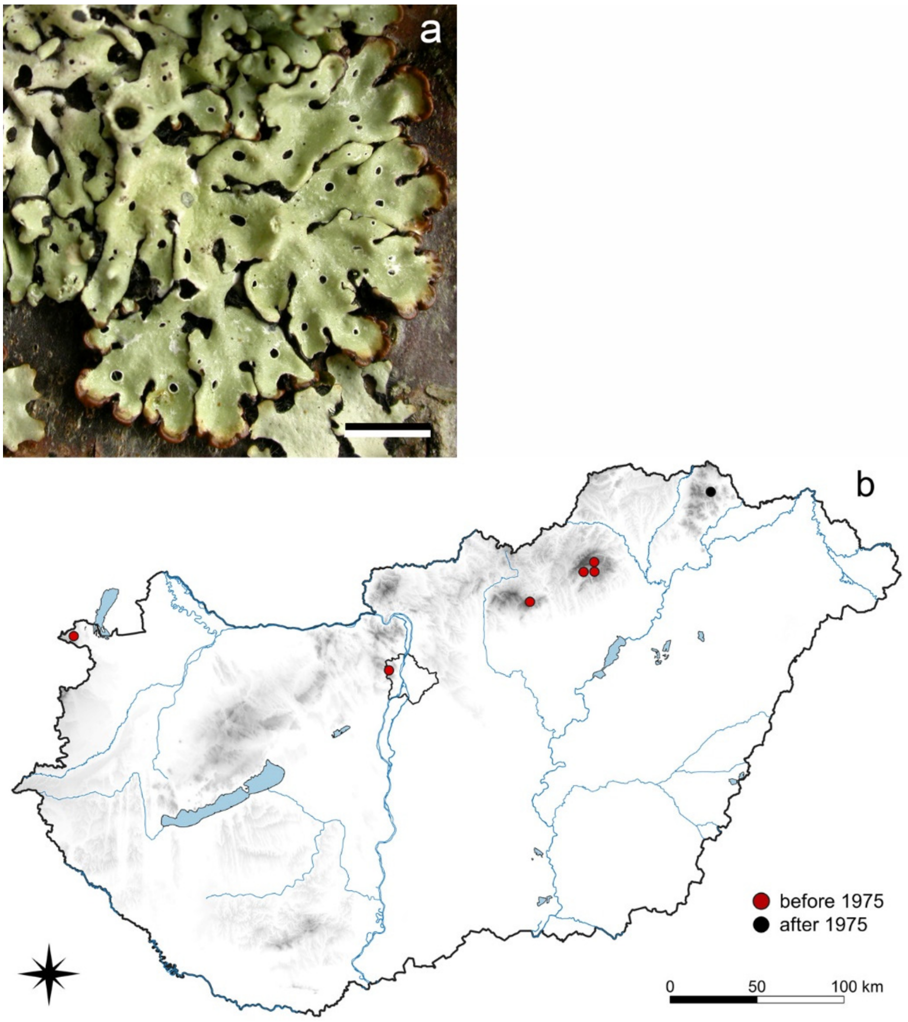

Figure S1. *Menegazzia terebrata* (**a**) habit (scale 1 cm); (**b**) its distribution in Hungary (10 records). Dots represent c. 5 × 6 km areas. (Photo: © E. Timdal)

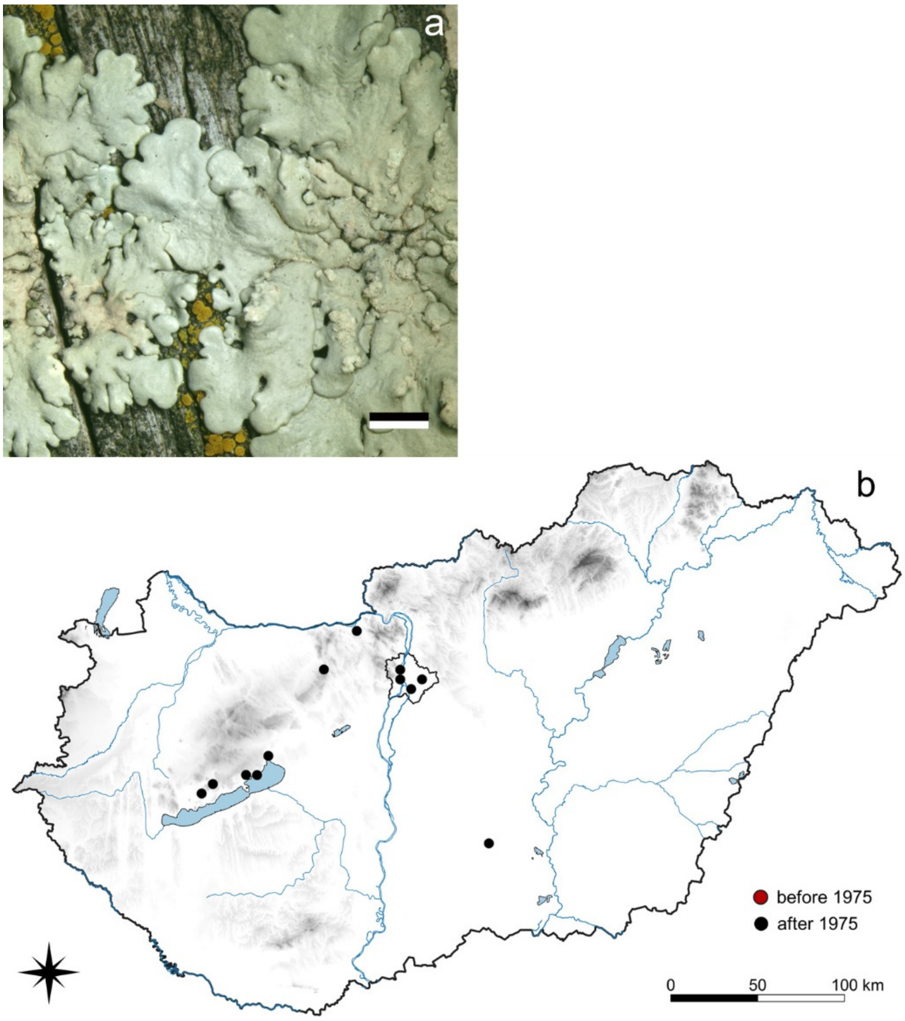

Figure S2. *Flavoparmelia soredians* (**a**) habit (scale 0.5 cm); (**b**) its distribution in Hungary (19 records). Dots represent c. 5 km × 6 km areas. (Photo: E. Farkas)

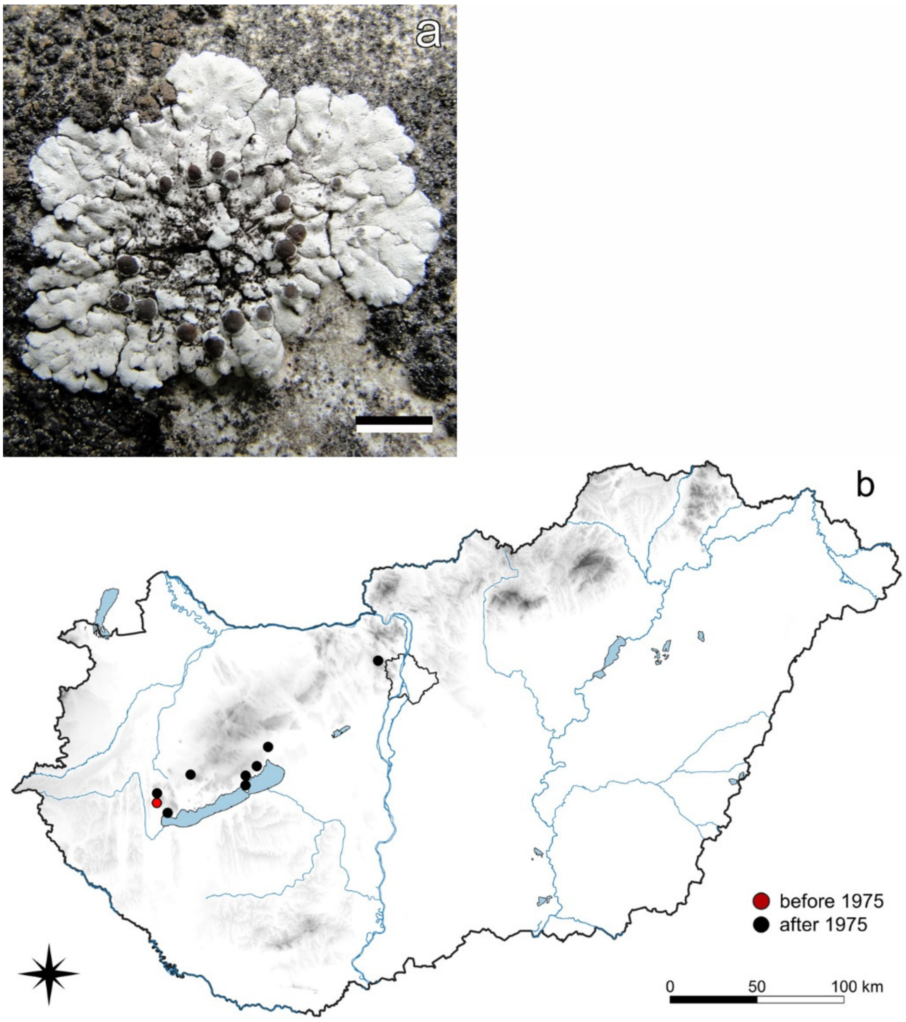

Figure S3. *Solenopsora candicans* (**a**) habit (scale 0.5 cm); (**b**) its distribution in Hungary (13 records). Dots represent c. 5 km × 6 km areas. (Photo: E. Farkas)

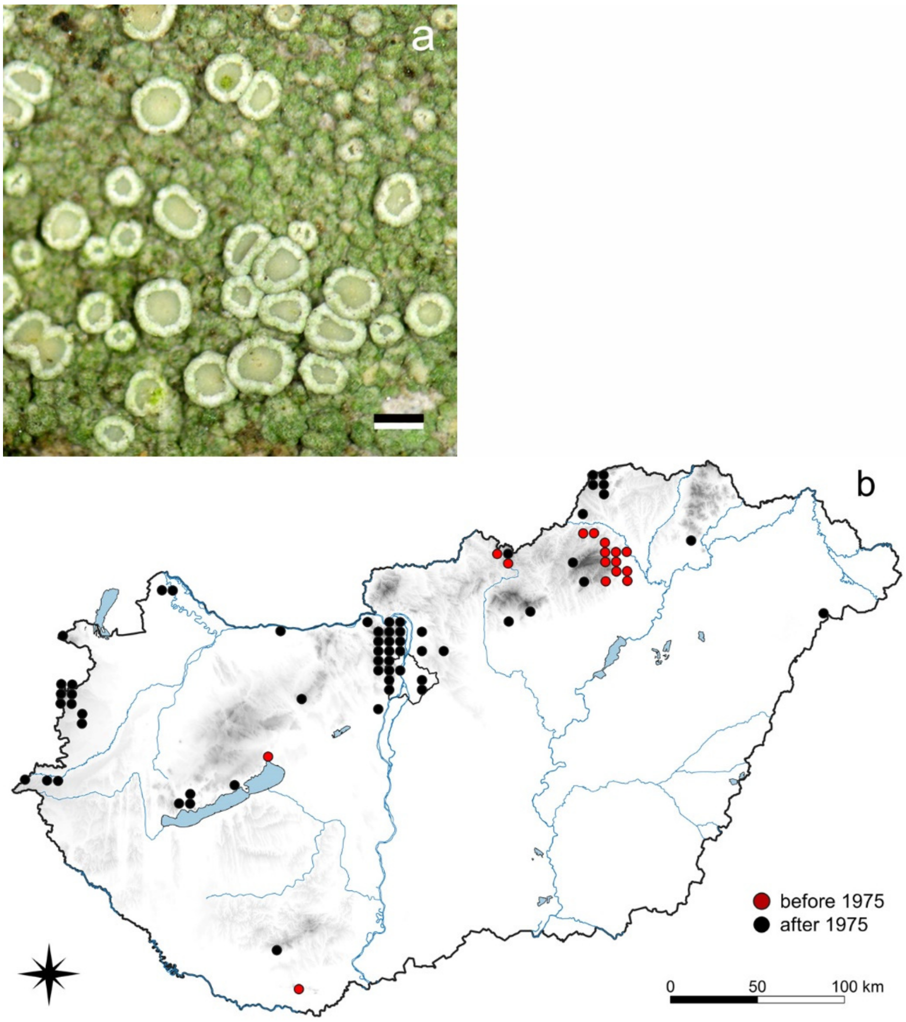

Figure S4. *Straminella conizaeoides* (**a**) habit (scale 0.1 cm); (**b**) its distribution in Hungary (241 records altogether). Dots represent c. 5 km × 6 km areas. (Photo: E. Farkas)

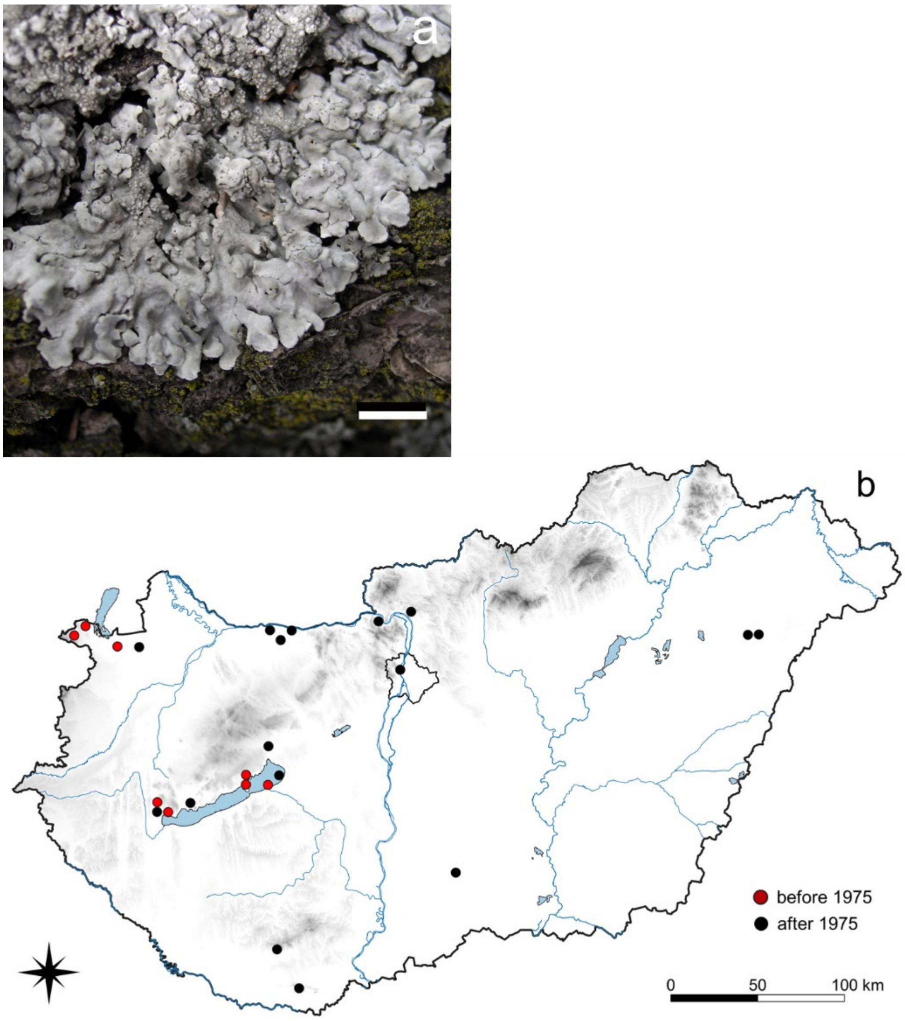

Figure S5. *Physcia aipolioides* (**a**) habit (scale 0.5 cm); (**b**) its distribution in Hungary (31 records). Dots represent c. 5 km × 6 km areas. (Photo: L. Lőkös)

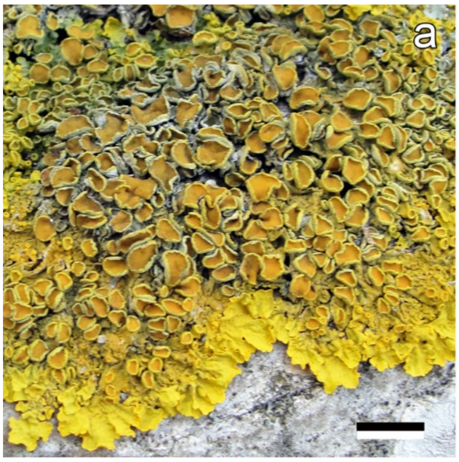

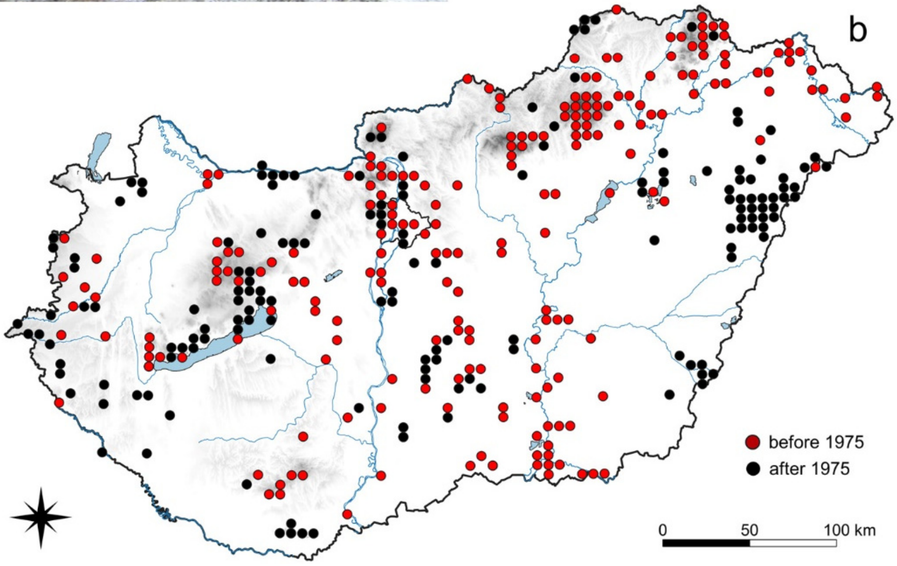

Figure S6. *Xanthoria parietina* (**a**) habit (scale 1 cm); (**b**) its distribution in Hungary (1023 records). Dots represent c. 5 km × 6 km areas. (Photo: E. Farkas)

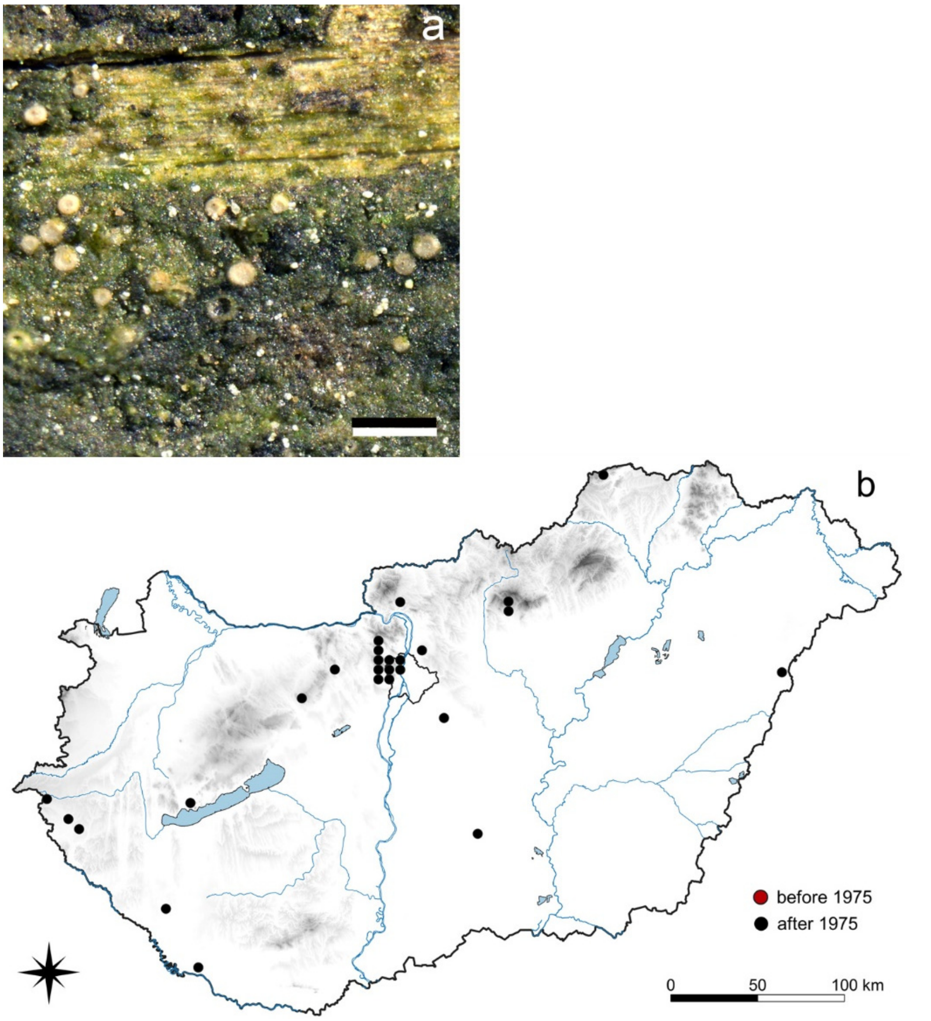

Figure S7. *Absconditella lignicola* (**a**) habit (scale 0.5 cm); (**b**) its distribution in Hungary (37 records, 2009–2022). Dots represent c. 5 km × 6 km areas. (Photo: E. Farkas)

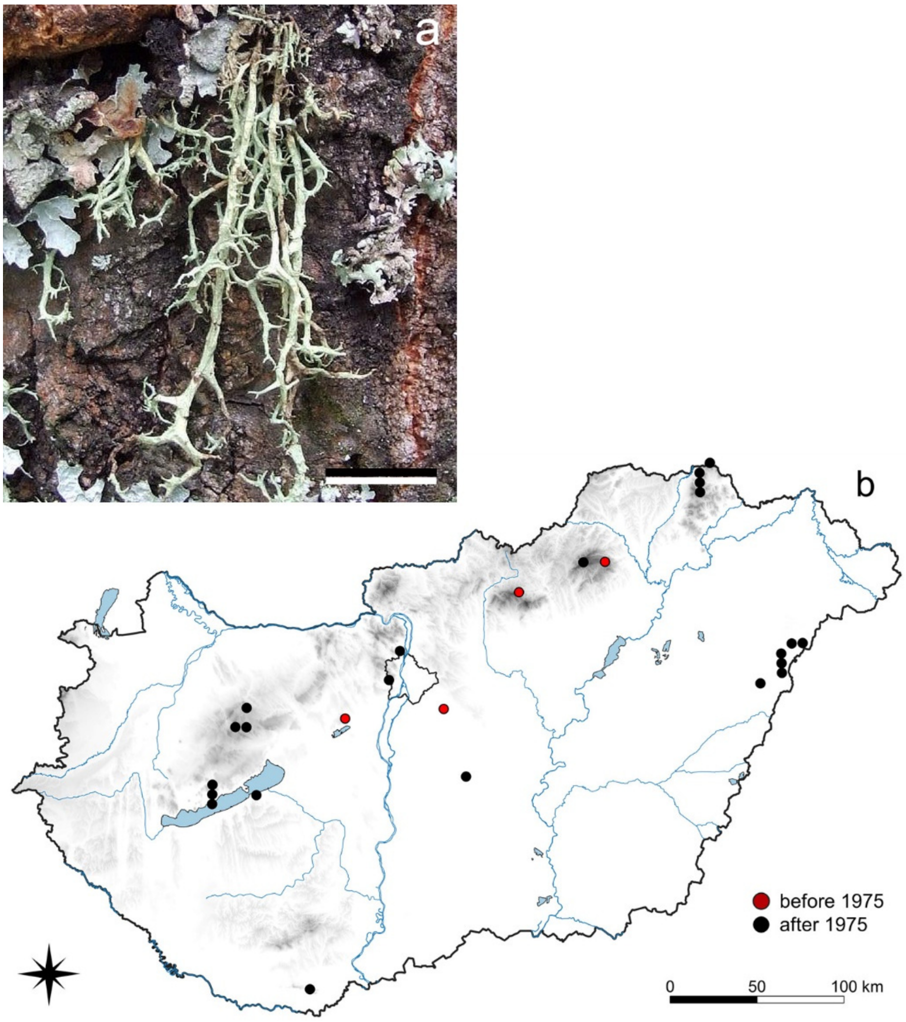

Figure S8. *Evernia divaricata* (**a**) habit (scale 1 cm); (**b**) its distribution in Hungary (35 records). Dots represent c. 5 km × 6 km areas. (Photo: G. Matus)

Table S1. Distribution records of illustrated species with main literature sources (containing published distribution records) and additional herbarium records (herbarium code, locality code [57,58], year)

Species name	Herbarium code	Locality code	Year
**Absconditella lignicola Vĕzda et Pišút**			
[72] Farkas, E.; Lőkös, L. (2021): Distribution of Absconditella lignicola (Stictidaceae, lichenized Ascomycetes) in Hungary. – Studia bot. hung. 52(2): 115–124. https://doi.org/10.17110/StudBot.2021.52.2.115			
**Coenogonium pineti (Ach.) Lücking et Lumbsch**			
Coenogonium pineti (Ach.) Lücking et Lumbsch	BP	9371.3	1954
Coenogonium pineti (Ach.) Lücking et Lumbsch	BP	8085.3	1974
Coenogonium pineti (Ach.) Lücking et Lumbsch	BP	8085.3	1981
Coenogonium pineti (Ach.) Lücking et Lumbsch	BP	0071.1	1988
Coenogonium pineti (Ach.) Lücking et Lumbsch	BP	9773.3	1988
Coenogonium pineti (Ach.) Lücking et Lumbsch	BP	8070.3	1991
Coenogonium pineti (Ach.) Lücking et Lumbsch	BP	8965.3	1993
Coenogonium pineti (Ach.) Lücking et Lumbsch	BP	9162.2	1993
Coenogonium pineti (Ach.) Lücking et Lumbsch	BP	9063.3	1994
Coenogonium pineti (Ach.) Lücking et Lumbsch	BP	0071.1	1996
Coenogonium pineti (Ach.) Lücking et Lumbsch	BP	8089.3	1998
Coenogonium pineti (Ach.) Lücking et Lumbsch	BP	9169.4	1998
Coenogonium pineti (Ach.) Lücking et Lumbsch	BP	7489.4	1998
Coenogonium pineti (Ach.) Lücking et Lumbsch	BP	9293.3	1998
Coenogonium pineti (Ach.) Lücking et Lumbsch	BP	7489.3	1999
Coenogonium pineti (Ach.) Lücking et Lumbsch	BP	8381.1	1999
Coenogonium pineti (Ach.) Lücking et Lumbsch	BP	8278.4	1999
Coenogonium pineti (Ach.) Lücking et Lumbsch	BP	7489.3	1999
Coenogonium pineti (Ach.) Lücking et Lumbsch	BP	9170.4	1999
Coenogonium pineti (Ach.) Lücking et Lumbsch	BP	8782.1	2000
Coenogonium pineti (Ach.) Lücking et Lumbsch	BP	7990.1	2000
Coenogonium pineti (Ach.) Lücking et Lumbsch	BP	0175.2	2000
Coenogonium pineti (Ach.) Lücking et Lumbsch	BP	8380.1	2000
Coenogonium pineti (Ach.) Lücking et Lumbsch	BP	7989.4	2001
Coenogonium pineti (Ach.) Lücking et Lumbsch	BP	8874.4	2001
Coenogonium pineti (Ach.) Lücking et Lumbsch	BP	8480.3	2001
Coenogonium pineti (Ach.) Lücking et Lumbsch	BP	7990.3	2001
Coenogonium pineti (Ach.) Lücking et Lumbsch	BP	8579.2	2002
Coenogonium pineti (Ach.) Lücking et Lumbsch	BP	7794.1	2002
Coenogonium pineti (Ach.) Lücking et Lumbsch	BP	8479.1	2002
Coenogonium pineti (Ach.) Lücking et Lumbsch	BP	8079.2	2002
Coenogonium pineti (Ach.) Lücking et Lumbsch	BP	8079.4	2002
Coenogonium pineti (Ach.) Lücking et Lumbsch	BP	7793.2	2002

Coenogonium pineti (Ach.) Lücking et Lumbsch	BP	8579.2	2002
Coenogonium pineti (Ach.) Lücking et Lumbsch	BP	9173.2	2003
Coenogonium pineti (Ach.) Lücking et Lumbsch	BP	8480.3	2003
Coenogonium pineti (Ach.) Lücking et Lumbsch	BP	7494.2	2004
Coenogonium pineti (Ach.) Lücking et Lumbsch	BP	9065.3	2004
Coenogonium pineti (Ach.) Lücking et Lumbsch	BP	7594.1	2004
Coenogonium pineti (Ach.) Lücking et Lumbsch	BP	7494.4	2004
Coenogonium pineti (Ach.) Lücking et Lumbsch	BP	9466.1	2005
Coenogonium pineti (Ach.) Lücking et Lumbsch	BP	8365.1	2005
Coenogonium pineti (Ach.) Lücking et Lumbsch	BP	8364.2	2005
Coenogonium pineti (Ach.) Lücking et Lumbsch	BP	7594.4	2006
Coenogonium pineti (Ach.) Lücking et Lumbsch	BP	7594.4	2006
Coenogonium pineti (Ach.) Lücking et Lumbsch	BP	8181.3	2007
Coenogonium pineti (Ach.) Lücking et Lumbsch	BP	8181.3	2007
Coenogonium pineti (Ach.) Lücking et Lumbsch	BP	8180.4	2007
Coenogonium pineti (Ach.) Lücking et Lumbsch	BP	8181.3	2007
Coenogonium pineti (Ach.) Lücking et Lumbsch	BP	8280.3	2008
Coenogonium pineti (Ach.) Lücking et Lumbsch	BP	8278.4	2008
Coenogonium pineti (Ach.) Lücking et Lumbsch	BP	8280.3	2008
Coenogonium pineti (Ach.) Lücking et Lumbsch	BP	8279.4	2008
Coenogonium pineti (Ach.) Lücking et Lumbsch	BP	8279.4	2008
Coenogonium pineti (Ach.) Lücking et Lumbsch	BP	9171.1	2009
Coenogonium pineti (Ach.) Lücking et Lumbsch	BP	9071.4	2009
Coenogonium pineti (Ach.) Lücking et Lumbsch	BP	9164.3	2009
Coenogonium pineti (Ach.) Lücking et Lumbsch	BP	8185.2	2009
Coenogonium pineti (Ach.) Lücking et Lumbsch	BP	8185.2	2009
Coenogonium pineti (Ach.) Lücking et Lumbsch	BP	7989.3	2010
Coenogonium pineti (Ach.) Lücking et Lumbsch	BP	8479.2	2011
Coenogonium pineti (Ach.) Lücking et Lumbsch	BP	8597.2	2013
Coenogonium pineti (Ach.) Lücking et Lumbsch	BP	8597.2	2013
Coenogonium pineti (Ach.) Lücking et Lumbsch	BP	8379.1	2014
Coenogonium pineti (Ach.) Lücking et Lumbsch	BP	9975.1	2014
Coenogonium pineti (Ach.) Lücking et Lumbsch	BP	8874.3	2014
Coenogonium pineti (Ach.) Lücking et Lumbsch	BMCRY	9072.4	2015
Coenogonium pineti (Ach.) Lücking et Lumbsch	BMCRY	8973.3	2015
Coenogonium pineti (Ach.) Lücking et Lumbsch	BMCRY	9072.2	2015
Coenogonium pineti (Ach.) Lücking et Lumbsch	BMCRY	8973.4	2015
Coenogonium pineti (Ach.) Lücking et Lumbsch	BP	8680.2	2015
Coenogonium pineti (Ach.) Lücking et Lumbsch	BP	8580.4	2015
Coenogonium pineti (Ach.) Lücking et Lumbsch	BMCRY	8773.1	2015
Coenogonium pineti (Ach.) Lücking et Lumbsch	BP	0071.1	2015
Coenogonium pineti (Ach.) Lücking et Lumbsch	BMCRY	9072.3	2015
Coenogonium pineti (Ach.) Lücking et Lumbsch	BMCRY	9073.1	2015
Coenogonium pineti (Ach.) Lücking et Lumbsch	BMCRY	8973.3	2015
Coenogonium pineti (Ach.) Lücking et Lumbsch	BMCRY	8974.1	2015
Coenogonium pineti (Ach.) Lücking et Lumbsch	BMCRY	9073.1	2015

Coenogonium pineti (Ach.) Lücking et Lumbsch	BMCRY	8973.3	2015
Coenogonium pineti (Ach.) Lücking et Lumbsch	BMCRY	8672.2	2016
Coenogonium pineti (Ach.) Lücking et Lumbsch	BMCRY	9171.1	2016
Coenogonium pineti (Ach.) Lücking et Lumbsch	BMCRY	9171.1	2016
Coenogonium pineti (Ach.) Lücking et Lumbsch	BMCRY	9071.4	2016
Coenogonium pineti (Ach.) Lücking et Lumbsch	BP	8773.3	2017
Coenogonium pineti (Ach.) Lücking et Lumbsch	BP	8179.2	2017
Coenogonium pineti (Ach.) Lücking et Lumbsch	BP	9465.2	2017
Coenogonium pineti (Ach.) Lücking et Lumbsch	BP	9567.1	2017
Coenogonium pineti (Ach.) Lücking et Lumbsch	BP	9367.3	2017
Coenogonium pineti (Ach.) Lücking et Lumbsch	BP	8380.2	2018
Coenogonium pineti (Ach.) Lücking et Lumbsch	BP	8673.3	2018
Coenogonium pineti (Ach.) Lücking et Lumbsch	BP	8782.1	2021
Coenogonium pineti (Ach.) Lücking et Lumbsch	BP	8479.4	2021
Coenogonium pineti (Ach.) Lücking et Lumbsch	BP	9365.1	2021
Coenogonium pineti (Ach.) Lücking et Lumbsch	BP	9265.3	2021
Coenogonium pineti (Ach.) Lücking et Lumbsch	BP	9365.1	2021
Coenogonium pineti (Ach.) Lücking et Lumbsch	BP	8180.1	2021
Coenogonium pineti (Ach.) Lücking et Lumbsch	BP	9170.4	2022
Coenogonium pineti (Ach.) Lücking et Lumbsch	BP	8482.1	2022
Coenogonium pineti (Ach.) Lücking et Lumbsch	BP	8477.3	2022
Coenogonium pineti (Ach.) Lücking et Lumbsch	BP	8479.2	2022

Evernia divaricata (L.) Ach.

Evernia divaricata (L.) Ach.	BP	9083.1	2009
Evernia divaricata (L.) Ach.	CBFS	9071.4	2009
Evernia divaricata (L.) Ach.	BP	8579.2	2001
Evernia divaricata (L.) Ach.	BP	7989.2	1932
Evernia divaricata (L.) Ach.	BP	7988.2	2006
Evernia divaricata (L.) Ach.	BP	9173.2	2003
Evernia divaricata (L.) Ach.	BP	8682.3	1925
Evernia divaricata (L.) Ach.	BP	8085.4	1925
Evernia divaricata (L.) Ach.	BP	8398.4	2012
Evernia divaricata (L.) Ach.	BP	8380.3	2012
Evernia divaricata (L.) Ach.	BP	8777.2	1866
Evernia divaricata (L.) Ach.	BP	0176.1	2000
Evernia divaricata (L.) Ach.	BP	7594.1	2006
Evernia divaricata (L.) Ach.	DE	8597.2	2013
Evernia divaricata (L.) Ach.	DE	8497.4	2013
Evernia divaricata (L.) Ach.	BMCRY	8673.3	2014
Evernia divaricata (L.) Ach.	BMCRY	9171.4	2016
Evernia divaricata (L.) Ach.	BMCRY	9171.4	2018
Evernia divaricata (L.) Ach.	BMCRY	9171.2	2016
Evernia divaricata (L.) Ach.	BMCRY	8773.3	2015

Evernia divaricata (L.) Ach.	BMCRY	8772.4	2018
Evernia divaricata (L.) Ach.	BMCRY	9071.4	2016
Evernia divaricata (L.) Ach.	DE	8398.3	2020
Evernia divaricata (L.) Ach.	DE	8398.4	2012
Evernia divaricata (L.) Ach.	DE	8398.4	2013
Evernia divaricata (L.) Ach.	DE	8596.4	2014
Evernia divaricata (L.) Ach.	DE	8497.4	2014
Evernia divaricata (L.) Ach.	DE	8497.4	2015
Evernia divaricata (L.) Ach.	DE	8497.4	2016
Evernia divaricata (L.) Ach.	DE	7594.3	2018
Evernia divaricata (L.) Ach.	DE	7494.2	2018
Evernia divaricata (L.) Ach.	DE	7494.2	2019
Evernia divaricata (L.) Ach.	DE	7494.3	2019
Evernia divaricata (L.) Ach.	DE	7494.2	2017
Evernia divaricata (L.) Ach.	DE	8497.2	2022

Flavoparmelia soredians (Nyl.) Hale

[65] Farkas, E.; Lajtha-Tabajdi, Á.; Lőkös, L.; Molnár, K.; Paczkó, L.; Sinigla, M. Flavoparmelia soredians (Parmeliaceae, lichenised Ascomycetes), a spreading lichen species in Hungary. Studia bot. hung. 2016, 47(1), 5–12. https://doi.org/10.17110/studbot.2016.47.1.5

Flavoparmelia soredians (Nyl.) Hale	BP	8476.4	2017
Flavoparmelia soredians (Nyl.) Hale	BP	9384.3	2020
Flavoparmelia soredians (Nyl.) Hale	BP	9569.2	2022
Flavoparmelia soredians (Nyl.) Hale	BP	8480.3	2022

Hyperphyscia adglutinata (Flörke) H. Mayrhofer et Poelt

Hyperphyscia adglutinata (Flörke) H. Mayrhofer et Poelt	BP	0176.2	1925
Hyperphyscia adglutinata (Flörke) H. Mayrhofer et Poelt	BP	9371.4	1954
Hyperphyscia adglutinata (Flörke) H. Mayrhofer et Poelt	BP	8772.1	1957
Hyperphyscia adglutinata (Flörke) H. Mayrhofer et Poelt	BP	9073.1	1960
Hyperphyscia adglutinata (Flörke) H. Mayrhofer et Poelt	BP	0176.1	2000
Hyperphyscia adglutinata (Flörke) H. Mayrhofer et Poelt	BP	9873.4	2008
Hyperphyscia adglutinata (Flörke) H. Mayrhofer et Poelt	BP	8480.3	2009
Hyperphyscia adglutinata (Flörke) H. Mayrhofer et Poelt	BP	9083.3	2009
Hyperphyscia adglutinata (Flörke) H. Mayrhofer et Poelt	BP	8966.3	2010
Hyperphyscia adglutinata (Flörke) H. Mayrhofer et Poelt	BP	8278.3	2011
Hyperphyscia adglutinata (Flörke) H. Mayrhofer et Poelt	BP	9073.1	2011
Hyperphyscia adglutinata (Flörke) H. Mayrhofer et Poelt	BP	8580.2	2011
Hyperphyscia adglutinata (Flörke) H. Mayrhofer et Poelt	BP	8580.2	2011
Hyperphyscia adglutinata (Flörke) H. Mayrhofer et Poelt	BP	8581.3	2011
Hyperphyscia adglutinata (Flörke) H. Mayrhofer et Poelt	BP	8581.3	2011

Hyperphyscia adglutinata (Flörke) H. Mayrhofer et Poelt	BP	9073.3	2012
Hyperphyscia adglutinata (Flörke) H. Mayrhofer et Poelt	BP	9185.2	2012
Hyperphyscia adglutinata (Flörke) H. Mayrhofer et Poelt	BP	8270.3	2012
Hyperphyscia adglutinata (Flörke) H. Mayrhofer et Poelt	BP	9974.2	2013
Hyperphyscia adglutinata (Flörke) H. Mayrhofer et Poelt	BP	8497.4	2013
Hyperphyscia adglutinata (Flörke) H. Mayrhofer et Poelt	BP	8498.2	2013
Hyperphyscia adglutinata (Flörke) H. Mayrhofer et Poelt	BP	8496.4	2013
Hyperphyscia adglutinata (Flörke) H. Mayrhofer et Poelt	BP	8597.3	2013
Hyperphyscia adglutinata (Flörke) H. Mayrhofer et Poelt	BP	8580.4	2013
Hyperphyscia adglutinata (Flörke) H. Mayrhofer et Poelt	JPU	9975.1	2014
Hyperphyscia adglutinata (Flörke) H. Mayrhofer et Poelt	BP	8480.3	2014
Hyperphyscia adglutinata (Flörke) H. Mayrhofer et Poelt	BP	8874.3	2014
Hyperphyscia adglutinata (Flörke) H. Mayrhofer et Poelt	BP	8381.3	2014
Hyperphyscia adglutinata (Flörke) H. Mayrhofer et Poelt	BP	9273.1	2014
Hyperphyscia adglutinata (Flörke) H. Mayrhofer et Poelt	BMCRY	8973.3	2015
Hyperphyscia adglutinata (Flörke) H. Mayrhofer et Poelt	BMCRY	8974.1	2015
Hyperphyscia adglutinata (Flörke) H. Mayrhofer et Poelt	BMCRY	9072.4	2015
Hyperphyscia adglutinata (Flörke) H. Mayrhofer et Poelt	BMCRY	9072.4	2015
Hyperphyscia adglutinata (Flörke) H. Mayrhofer et Poelt	BMCRY	9171.1	2016
Hyperphyscia adglutinata (Flörke) H. Mayrhofer et Poelt	BP	9680.2	2016
Hyperphyscia adglutinata (Flörke) H. Mayrhofer et Poelt	BP	8580.1	2016
Hyperphyscia adglutinata (Flörke) H. Mayrhofer et Poelt	BP	9580.4	2016
Hyperphyscia adglutinata (Flörke) H. Mayrhofer et Poelt	BP	9680.2	2016
Hyperphyscia adglutinata (Flörke) H. Mayrhofer et Poelt	BP	8579.2	2017
Hyperphyscia adglutinata (Flörke) H. Mayrhofer et Poelt	BP	9367.3	2017
Hyperphyscia adglutinata (Flörke) H. Mayrhofer et Poelt	BP	8479.4	2017
Hyperphyscia adglutinata (Flörke) H. Mayrhofer et Poelt	BP	9171.1	2017
Hyperphyscia adglutinata (Flörke) H. Mayrhofer et Poelt	BP	8480.3	2017
Hyperphyscia adglutinata (Flörke) H. Mayrhofer et Poelt	BP	8782.1	2021
Hyperphyscia adglutinata (Flörke) H. Mayrhofer et Poelt	BP	8479.2	2021
Hyperphyscia adglutinata (Flörke) H. Mayrhofer et Poelt	BP	8782.1	2021
Hyperphyscia adglutinata (Flörke) H. Mayrhofer et Poelt	BP	8580.2	2021
Hyperphyscia adglutinata (Flörke) H. Mayrhofer et Poelt	BP	8782.1	2021
Hyperphyscia adglutinata (Flörke) H. Mayrhofer et Poelt	BP	0176.2	2021
Hyperphyscia adglutinata (Flörke) H. Mayrhofer et Poelt	BP	9769.1	2021
Hyperphyscia adglutinata (Flörke) H. Mayrhofer et Poelt	BP	9265.3	2021
Hyperphyscia adglutinata (Flörke) H. Mayrhofer et Poelt	BP	0176.2	2021
Hyperphyscia adglutinata (Flörke) H. Mayrhofer et Poelt	BP	9383.2	2021
Hyperphyscia adglutinata (Flörke) H. Mayrhofer et Poelt	BP	9170.4	2022
Hyperphyscia adglutinata (Flörke) H. Mayrhofer et Poelt	BP	8880.3	2019
Hyperphyscia adglutinata (Flörke) H. Mayrhofer et Poelt	BMCRY	8280.4	2018

Lobaria pulmonaria L.

[63] Farkas, E.; Lőkös, L. Lobaria pulmonaria (lichen-forming fungi) in Hungary. (A tüdőzuzmó (Lobaria pulmonaria) elterjedése Magyarországon). Mikol. Közlem., Clusiana 2009, 48(1), 11–18.

Lobaria pulmonaria L.	BP	7989.3	2016
Lobaria pulmonaria L.	BP	7989.3	2016

Menegazzia terebrata (Hoffm.) A. Massal.

[49] Verseghy, K. Magyarország zuzmóflórájának kézikönyve. Magyar Természettudományi Múzeum: Budapest, Hungary, 1994; pp. 1–415.

Menegazzia terebrata (Hoffm.) A. Massal.	BP	7594.4	1997

Physcia aipolioides (Nádv.) Breuss et Türk

[71] Lisická, E.; Lackovičová, A.; Liška, J.; Lőkös, L.; Lisický, M.J. Physcia aipolioides – ein Beispiel einer invasiven Flechte oder einer unterschätzen Verbreitung? (Physcia aipolioides – an example of an invasive lichen or an underestimated distribution?). Sauteria 2008, 15, 303–318.

Physcia aipolioides (Nádv.) Breuss et Türk	BP	9073.3	1954
Physcia aipolioides (Nádv.) Breuss et Türk	BP	9073.3	1954
Physcia aipolioides (Nádv.) Breuss et Türk	BP	9269.1	1957
Physcia aipolioides (Nádv.) Breuss et Türk	BP	9074.3	1958
Physcia aipolioides (Nádv.) Breuss et Türk	BP	9074.3	1958
Physcia aipolioides (Nádv.) Breuss et Türk	BP	8365.1	1961
Physcia aipolioides (Nádv.) Breuss et Türk	BP	8265.4	1961
Physcia aipolioides (Nádv.) Breuss et Türk	BP	8367.3	1961
Physcia aipolioides (Nádv.) Breuss et Türk	BP	9169.3	1961
Physcia aipolioides (Nádv.) Breuss et Türk	BP	9269.2	1961
Physcia aipolioides (Nádv.) Breuss et Türk	BP	9073.1	1961
Physcia aipolioides (Nádv.) Breuss et Türk	BP	8365.1	1963
Physcia aipolioides (Nádv.) Breuss et Türk	BP	9073.1	1964
Physcia aipolioides (Nádv.) Breuss et Türk	BP	8279.1	1986
Physcia aipolioides (Nádv.) Breuss et Türk	BP	8374.2	1999
Physcia aipolioides (Nádv.) Breuss et Türk	BP	8374.2	1999
Physcia aipolioides (Nádv.) Breuss et Türk	BP	8275.3	1999
Physcia aipolioides (Nádv.) Breuss et Türk	BP	8274.3	1999
Physcia aipolioides (Nádv.) Breuss et Türk	BP	0175.2	2000
Physcia aipolioides (Nádv.) Breuss et Türk	BP	9269.1	2003
Physcia aipolioides (Nádv.) Breuss et Türk	BP	8874.3	2007
Physcia aipolioides (Nádv.) Breuss et Türk	BP	8180.4	2007
Physcia aipolioides (Nádv.) Breuss et Türk	BP	8396.2	2013
Physcia aipolioides (Nádv.) Breuss et Türk	DE	8396.1	2013

Physcia aipolioides (Nádv.) Breuss et Türk	BP	9074.2	2015
Physcia aipolioides (Nádv.) Breuss et Türk	BP	8368.3	2022
Physcia aipolioides (Nádv.) Breuss et Türk	BP	8480.3	2022
Physcia aipolioides (Nádv.) Breuss et Türk	BP	8480.3	2022
Physcia aipolioides (Nádv.) Breuss et Türk	BP	9974.2	2013
Physcia aipolioides (Nádv.) Breuss et Türk	BP	9582.2	2012
Physcia aipolioides (Nádv.) Breuss et Türk	BP	9170.4	2014

Piccolia ochrophora (Nyl.) Hafellner

Piccolia ochrophora (Nyl.) Hafellner	BP	8379.1	2014
Piccolia ochrophora (Nyl.) Hafellner	BP	8480.3	2013
Piccolia ochrophora (Nyl.) Hafellner	BP	8378.2	2014
Piccolia ochrophora (Nyl.) Hafellner	BP	8580.4	2004
Piccolia ochrophora (Nyl.) Hafellner	VBI, S	9182.2	1987
Piccolia ochrophora (Nyl.) Hafellner	CBFS	7988.2	2006
Piccolia ochrophora (Nyl.) Hafellner	BP	8278.3	2011
Piccolia ochrophora (Nyl.) Hafellner	BP	8096.1	2010
Piccolia ochrophora (Nyl.) Hafellner	BP	9171.1	2009
Piccolia ochrophora (Nyl.) Hafellner	BP	8381.3	2014
Piccolia ochrophora (Nyl.) Hafellner	BMCRY	8476.4	2016
Piccolia ochrophora (Nyl.) Hafellner	BMCRY	8380.4	2018
Piccolia ochrophora (Nyl.) Hafellner	BP	8498.2	2013
Piccolia ochrophora (Nyl.) Hafellner	BP	8680.2	2013
Piccolia ochrophora (Nyl.) Hafellner	BP	8580.4	2013
Piccolia ochrophora (Nyl.) Hafellner	BP	9367.3	2017

Scoliciosporum chlorococcum (Stenh.) Vězda

[49] Verseghy, K. Magyarország zuzmóflórájának kézikönyve. Magyar Természettudományi Múzeum: Budapest, Hungary, 1994; pp. 1–415.

[70] Lőkös, L. A Bacidia s. l. zuzmónemzetség hazai fajainak taxonómiai revíziója. [Taxonomic revision of the Bacidia s. l. species in Hungary.] PhD Thesis, PTE, Pécs, Hungary, 2005; pp. 1–158.

Scoliciosporum chlorococcum (Stenh.) Vězda	BMCRY	9171.2	2013
Scoliciosporum chlorococcum (Stenh.) Vězda	BMCRY	8773.3	2013
Scoliciosporum chlorococcum (Stenh.) Vězda	BMCRY	9170.3	2014
Scoliciosporum chlorococcum (Stenh.) Vězda	BMCRY	8572.1	2014
Scoliciosporum chlorococcum (Stenh.) Vězda	BMCRY	9072.3	2015
Scoliciosporum chlorococcum (Stenh.) Vězda	BMCRY	9072.4	2015
Scoliciosporum chlorococcum (Stenh.) Vězda	BMCRY	9072.2	2015
Scoliciosporum chlorococcum (Stenh.) Vězda	BMCRY	9071.4	2016
Scoliciosporum chlorococcum (Stenh.) Vězda	BMCRY	9171.1	2016
Scoliciosporum chlorococcum (Stenh.) Vězda	BMCRY	9171.2	2016
Scoliciosporum chlorococcum (Stenh.) Vězda	BMCRY	9171.1	2016

Solenopsora candicans (Dicks.) J. Steiner

[69] Farkas, E.; Guttová, A.; Lőkös, L.; Molnár, K. Distribution of Solenopsora candicans (lichen-forming fungi, Catillariaceae) in Hungary. Acta Bot. Hung. 2011, 53(3–4), 305–311. https://doi.org/10.1556/abot.53.2011.3-4.12

Solenopsora candicans (Dicks.) J. Steiner	BMCRY	9070.2	2015

Straminella conizaeoides (Cromb.) S. Y. Kondr., Lőkös et Farkas

[49] Verseghy, K. Magyarország zuzmóflórájának kézikönyve. Magyar Természettudományi Múzeum: Budapest, Hungary, 1994; pp. 1–415.

Straminella conizaeoides (Cromb.) S. Y. Kondr., Lőkös et Farkas	BP	7688.4	1995
Straminella conizaeoides (Cromb.) S. Y. Kondr., Lőkös et Farkas	BP	8285.1	1996
Straminella conizaeoides (Cromb.) S. Y. Kondr., Lőkös et Farkas	BP	8974.1	1997
Straminella conizaeoides (Cromb.) S. Y. Kondr., Lőkös et Farkas	BP	9170.4	1997
Straminella conizaeoides (Cromb.) S. Y. Kondr., Lőkös et Farkas	BP	8274.2	1998
Straminella conizaeoides (Cromb.) S. Y. Kondr., Lőkös et Farkas	BP	7589.1	1998
Straminella conizaeoides (Cromb.) S. Y. Kondr., Lőkös et Farkas	BP	8274.2	1998
Straminella conizaeoides (Cromb.) S. Y. Kondr., Lőkös et Farkas	BP	9170.2	1999
Straminella conizaeoides (Cromb.) S. Y. Kondr., Lőkös et Farkas	BP	8274.2	1999
Straminella conizaeoides (Cromb.) S. Y. Kondr., Lőkös et Farkas	BP	8480.3	1999
Straminella conizaeoides (Cromb.) S. Y. Kondr., Lőkös et Farkas	BP	8274.2	1999
Straminella conizaeoides (Cromb.) S. Y. Kondr., Lőkös et Farkas	BP	9170.3	1999
Straminella conizaeoides (Cromb.) S. Y. Kondr., Lőkös et Farkas	BP	7589.1	2000
Straminella conizaeoides (Cromb.) S. Y. Kondr., Lőkös et Farkas	BP	0175.2	2000
Straminella conizaeoides (Cromb.) S. Y. Kondr., Lőkös et Farkas	BP	8480.3	2000
Straminella conizaeoides (Cromb.) S. Y. Kondr., Lőkös et Farkas	BP	8281.3	2000
Straminella conizaeoides (Cromb.) S. Y. Kondr., Lőkös et Farkas	BP	8379.3	2001
Straminella conizaeoides (Cromb.) S. Y. Kondr., Lőkös et Farkas	BP	8382.3	2001
Straminella conizaeoides (Cromb.) S. Y. Kondr., Lőkös et Farkas	EGR	7884.4	2001
Straminella conizaeoides (Cromb.) S. Y. Kondr., Lőkös et Farkas	BP	8379.3	2001
Straminella conizaeoides (Cromb.) S. Y. Kondr., Lőkös et Farkas	BP	8579.2	2002
Straminella conizaeoides (Cromb.) S. Y. Kondr., Lőkös et Farkas	BP	8479.4	2002
Straminella conizaeoides (Cromb.) S. Y. Kondr., Lőkös et Farkas	BP	8479.4	2002
Straminella conizaeoides (Cromb.) S. Y. Kondr., Lőkös et Farkas	EGR	7988.1	2003
Straminella conizaeoides (Cromb.) S. Y. Kondr., Lőkös et Farkas	EGR	8186.3	2004
Straminella conizaeoides (Cromb.) S. Y. Kondr., Lőkös et Farkas	EGR	8088.2	2004

Xanthoria parietina (L.) Th. Fr.

[49] Verseghy, K. Magyarország zuzmóflórájának kézikönyve. Magyar Természettudományi Múzeum: Budapest, Hungary, 1994; pp. 1–415.

Xanthoria parietina (L.) Th. Fr.	BP	8299.3	1997
Xanthoria parietina (L.) Th. Fr.	BP	8275.3	1998
Xanthoria parietina (L.) Th. Fr.	BP	8275.3	1998
Xanthoria parietina (L.) Th. Fr.	BP	8275.3	1998
Xanthoria parietina (L.) Th. Fr.	BP	8274.4	1998
Xanthoria parietina (L.) Th. Fr.	BP	8274.4	1998
Xanthoria parietina (L.) Th. Fr.	BP	8274.4	1998
Xanthoria parietina (L.) Th. Fr.	BP	8274.4	1998
Xanthoria parietina (L.) Th. Fr.	BP	8274.4	1998
Xanthoria parietina (L.) Th. Fr.	BP	8274.4	1998
Xanthoria parietina (L.) Th. Fr.	BP	8274.4	1998
Xanthoria parietina (L.) Th. Fr.	BP	8274.4	1998
Xanthoria parietina (L.) Th. Fr.	BP	8274.1	1998
Xanthoria parietina (L.) Th. Fr.	BP	8274.3	1998
Xanthoria parietina (L.) Th. Fr.	BP	8374.2	1998
Xanthoria parietina (L.) Th. Fr.	BP	8374.2	1998
Xanthoria parietina (L.) Th. Fr.	BP	9293.1	1998
Xanthoria parietina (L.) Th. Fr.	BP	9394.3	1998
Xanthoria parietina (L.) Th. Fr.	BP	9394.3	1998
Xanthoria parietina (L.) Th. Fr.	BP	9294.3	1998
Xanthoria parietina (L.) Th. Fr.	BP	9294.3	1998
Xanthoria parietina (L.) Th. Fr.	BP	9294.3	1998
Xanthoria parietina (L.) Th. Fr.	BP	9294.3	1998
Xanthoria parietina (L.) Th. Fr.	BP	9394.2	1998
Xanthoria parietina (L.) Th. Fr.	BP	9394.2	1998
Xanthoria parietina (L.) Th. Fr.	BP	9394.2	1998
Xanthoria parietina (L.) Th. Fr.	BP	9394.1	1998
Xanthoria parietina (L.) Th. Fr.	BP	9394.1	1998
Xanthoria parietina (L.) Th. Fr.	BP	9394.1	1998
Xanthoria parietina (L.) Th. Fr.	BP	9394.1	1998
Xanthoria parietina (L.) Th. Fr.	BP	9394.1	1998
Xanthoria parietina (L.) Th. Fr.	BP	9294.3	1998
Xanthoria parietina (L.) Th. Fr.	BP	9293.4	1998
Xanthoria parietina (L.) Th. Fr.	BP	9293.4	1998
Xanthoria parietina (L.) Th. Fr.	BP	0175.2	1999
Xanthoria parietina (L.) Th. Fr.	BP	0176.1	1999
Xanthoria parietina (L.) Th. Fr.	BP	0175.2	1999
Xanthoria parietina (L.) Th. Fr.	BP	0176.1	1999
Xanthoria parietina (L.) Th. Fr.	BP	0175.2	1999
Xanthoria parietina (L.) Th. Fr.	BP	9293.4	1999
Xanthoria parietina (L.) Th. Fr.	BP	7588.2	1999
Xanthoria parietina (L.) Th. Fr.	BP	0176.1	1999
Xanthoria parietina (L.) Th. Fr.	BP	0175.2	1999

Xanthoria parietina (L.) Th. Fr.	BP	0175.2	1999
Xanthoria parietina (L.) Th. Fr.	BP	8675.1	2000
Xanthoria parietina (L.) Th. Fr.	BP	9767.1	2000
Xanthoria parietina (L.) Th. Fr.	BP	0175.2	2000
Xanthoria parietina (L.) Th. Fr.	BP	0176.1	2000
Xanthoria parietina (L.) Th. Fr.	BP	0176.1	2000
Xanthoria parietina (L.) Th. Fr.	BP	0176.1	2000
Xanthoria parietina (L.) Th. Fr.	BP	0176.2	2000
Xanthoria parietina (L.) Th. Fr.	BP	0175.2	2000
Xanthoria parietina (L.) Th. Fr.	BP	0175.2	2000
Xanthoria parietina (L.) Th. Fr.	BP	0175.2	2000
Xanthoria parietina (L.) Th. Fr.	BP	7788.4	2005
Xanthoria parietina (L.) Th. Fr.	BP	8180.4	2007
Xanthoria parietina (L.) Th. Fr.	BP	9873.4	2008
Xanthoria parietina (L.) Th. Fr.	BP	9873.4	2008
Xanthoria parietina (L.) Th. Fr.	BP	9873.4	2008
Xanthoria parietina (L.) Th. Fr.	BP	9873.4	2008
Xanthoria parietina (L.) Th. Fr.	BP	9071.4	2009
Xanthoria parietina (L.) Th. Fr.	BP	9171.1	2009
Xanthoria parietina (L.) Th. Fr.	BP	8096.1	2010
Xanthoria parietina (L.) Th. Fr.	BP	7996.3	2010
Xanthoria parietina (L.) Th. Fr.	BP	7996.3	2010
Xanthoria parietina (L.) Th. Fr.	BP	8966.3	2010
Xanthoria parietina (L.) Th. Fr.	BP	9182.2	2011
Xanthoria parietina (L.) Th. Fr.	BP	8299.2	2011
Xanthoria parietina (L.) Th. Fr.	BP	8299.2	2011
Xanthoria parietina (L.) Th. Fr.	BP	8299.2	2011
Xanthoria parietina (L.) Th. Fr.	BP	8278.4	2011
Xanthoria parietina (L.) Th. Fr.	BP	8278.4	2011
Xanthoria parietina (L.) Th. Fr.	BP	8278.4	2011
Xanthoria parietina (L.) Th. Fr.	BP	8278.4	2011
Xanthoria parietina (L.) Th. Fr.	BP	9185.2	2012
Xanthoria parietina (L.) Th. Fr.	BP, VBI	9073.2	2012
Xanthoria parietina (L.) Th. Fr.	VBI	8368.1	2012
Xanthoria parietina (L.) Th. Fr.	BMCRY	9073.1	2012
Xanthoria parietina (L.) Th. Fr.	BMCRY	8974.1	2012
Xanthoria parietina (L.) Th. Fr.	BMCRY	8873.2	2012
Xanthoria parietina (L.) Th. Fr.	BP	8496.2	2012
Xanthoria parietina (L.) Th. Fr.	BP	9582.2	2012
Xanthoria parietina (L.) Th. Fr.	BP	9582.2	2012
Xanthoria parietina (L.) Th. Fr.	BP	9270.1	2013
Xanthoria parietina (L.) Th. Fr.	BP	8597.2	2013
Xanthoria parietina (L.) Th. Fr.	BP	8497.3	2013
Xanthoria parietina (L.) Th. Fr.	BP	8498.1	2013
Xanthoria parietina (L.) Th. Fr.	BP	8497.2	2013
Xanthoria parietina (L.) Th. Fr.	BP, DE	8496.2	2013

Xanthoria parietina (L.) Th. Fr.	BP, DE	8497.4	2013
Xanthoria parietina (L.) Th. Fr.	BP, VBI	8873.3	2013
Xanthoria parietina (L.) Th. Fr.	BP, VBI	8873.3	2013
Xanthoria parietina (L.) Th. Fr.	VBI	8368.4	2013
Xanthoria parietina (L.) Th. Fr.	VBI	8368.4	2013
Xanthoria parietina (L.) Th. Fr.	VBI	8368.2	2013
Xanthoria parietina (L.) Th. Fr.	VBI	8368.4	2013
Xanthoria parietina (L.) Th. Fr.	VBI	9073.3	2013
Xanthoria parietina (L.) Th. Fr.	VBI	8467.2	2013
Xanthoria parietina (L.) Th. Fr.	BMCRY	8574.3	2013
Xanthoria parietina (L.) Th. Fr.	BMCRY	8974.2	2013
Xanthoria parietina (L.) Th. Fr.	BMCRY	8974.2	2013
Xanthoria parietina (L.) Th. Fr.	BMCRY	8974.2	2013
Xanthoria parietina (L.) Th. Fr.	BMCRY	8672.2	2013
Xanthoria parietina (L.) Th. Fr.	BMCRY	8781.1	2013
Xanthoria parietina (L.) Th. Fr.	BMCRY	8773.3	2013
Xanthoria parietina (L.) Th. Fr.	BMCRY	8974.2	2013
Xanthoria parietina (L.) Th. Fr.	BMCRY	8874.3	2013
Xanthoria parietina (L.) Th. Fr.	BMCRY	8874.3	2013
Xanthoria parietina (L.) Th. Fr.	BMCRY	9274.2	2013
Xanthoria parietina (L.) Th. Fr.	BMCRY	8971.3	2013
Xanthoria parietina (L.) Th. Fr.	BMCRY	8873.4	2013
Xanthoria parietina (L.) Th. Fr.	BMCRY	9468.2	2013
Xanthoria parietina (L.) Th. Fr.	BMCRY	9468.2	2013
Xanthoria parietina (L.) Th. Fr.	BMCRY	9469.1	2013
Xanthoria parietina (L.) Th. Fr.	BMCRY	9171.2	2013
Xanthoria parietina (L.) Th. Fr.	BP	8597.2	2013
Xanthoria parietina (L.) Th. Fr.	BP	8597.2	2013
Xanthoria parietina (L.) Th. Fr.	BP	8498.1	2013
Xanthoria parietina (L.) Th. Fr.	BP	8498.1	2013
Xanthoria parietina (L.) Th. Fr.	BP	8497.2	2013
Xanthoria parietina (L.) Th. Fr.	BP	8496.2	2013
Xanthoria parietina (L.) Th. Fr.	BP	8496.2	2013
Xanthoria parietina (L.) Th. Fr.	BP	8496.2	2013
Xanthoria parietina (L.) Th. Fr.	BP	8496.4	2013
Xanthoria parietina (L.) Th. Fr.	BP	8496.4	2013
Xanthoria parietina (L.) Th. Fr.	BP	8498.2	2013
Xanthoria parietina (L.) Th. Fr.	BP	8498.2	2013
Xanthoria parietina (L.) Th. Fr.	BP	8498.1	2013
Xanthoria parietina (L.) Th. Fr.	BP	8498.1	2013
Xanthoria parietina (L.) Th. Fr.	BP	8497.4	2013
Xanthoria parietina (L.) Th. Fr.	BP	8396.1	2013
Xanthoria parietina (L.) Th. Fr.	BP	8396.2	2013
Xanthoria parietina (L.) Th. Fr.	BMCRY	9171.2	2013
Xanthoria parietina (L.) Th. Fr.	BMCRY	9171.2	2013
Xanthoria parietina (L.) Th. Fr.	BMCRY	9171.2	2013

Xanthoria parietina (L.) Th. Fr.	BMCRY	9171.2	2013
Xanthoria parietina (L.) Th. Fr.	BMCRY	9171.2	2013
Xanthoria parietina (L.) Th. Fr.	BP	8680.2	2013
Xanthoria parietina (L.) Th. Fr.	BP	8680.2	2013
Xanthoria parietina (L.) Th. Fr.	BP	8680.2	2013
Xanthoria parietina (L.) Th. Fr.	BP	8580.4	2013
Xanthoria parietina (L.) Th. Fr.	BP	8680.2	2013
Xanthoria parietina (L.) Th. Fr.	BP	8580.4	2013
Xanthoria parietina (L.) Th. Fr.	BP	8874.3	2014
Xanthoria parietina (L.) Th. Fr.	BP	9170.4	2014
Xanthoria parietina (L.) Th. Fr.	BP	9170.4	2014
Xanthoria parietina (L.) Th. Fr.	BP	9170.4	2014
Xanthoria parietina (L.) Th. Fr.	BP	9170.3	2014
Xanthoria parietina (L.) Th. Fr.	BMCRY	8974.1	2015
Xanthoria parietina (L.) Th. Fr.	BMCRY	8973.4	2015
Xanthoria parietina (L.) Th. Fr.	BMCRY	8973.1	2015
Xanthoria parietina (L.) Th. Fr.	BP	9478.4	2016
Xanthoria parietina (L.) Th. Fr.	BP	8495.2	2016
Xanthoria parietina (L.) Th. Fr.	BMCRY	9680.2	2016
Xanthoria parietina (L.) Th. Fr.	BP	9680.2	2016
Xanthoria parietina (L.) Th. Fr.	BP	9680.2	2016
Xanthoria parietina (L.) Th. Fr.	BP	9680.2	2016
Xanthoria parietina (L.) Th. Fr.	BP	9680.2	2016
Xanthoria parietina (L.) Th. Fr.	BP	9680.2	2016
Xanthoria parietina (L.) Th. Fr.	BP	9680.2	2016
Xanthoria parietina (L.) Th. Fr.	BP	9680.2	2016
Xanthoria parietina (L.) Th. Fr.	BP	9580.4	2016
Xanthoria parietina (L.) Th. Fr.	BP	9580.4	2016
Xanthoria parietina (L.) Th. Fr.	BP	9580.4	2016
Xanthoria parietina (L.) Th. Fr.	BMCRY	8476.4	2017
Xanthoria parietina (L.) Th. Fr.	BP	8773.3	2017
Xanthoria parietina (L.) Th. Fr.	BP	9367.3	2017
Xanthoria parietina (L.) Th. Fr.	BP	9367.3	2017
Xanthoria parietina (L.) Th. Fr.	BP	9467.3	2017
Xanthoria parietina (L.) Th. Fr.	BP	9465.2	2017
Xanthoria parietina (L.) Th. Fr.	BP	8579.2	2017
Xanthoria parietina (L.) Th. Fr.	BMCRY	8476.4	2017
Xanthoria parietina (L.) Th. Fr.	BP	8476.4	2017
Xanthoria parietina (L.) Th. Fr.	BP	8476.4	2017
Xanthoria parietina (L.) Th. Fr.	BP	8476.4	2017
Xanthoria parietina (L.) Th. Fr.	BP	8476.4	2017
Xanthoria parietina (L.) Th. Fr.	BP	9383.2	2018
Xanthoria parietina (L.) Th. Fr.	BP	8294.4	2018
Xanthoria parietina (L.) Th. Fr.	BP	8380.2	2018
Xanthoria parietina (L.) Th. Fr.	BP	8380.2	2018
Xanthoria parietina (L.) Th. Fr.	BP	8380.4	2018

Xanthoria parietina (L.) Th. Fr.	BP	8380.4	2018
Xanthoria parietina (L.) Th. Fr.	BP	8380.4	2018
Xanthoria parietina (L.) Th. Fr.	BP	8380.4	2018
Xanthoria parietina (L.) Th. Fr.	BP	8187.1	2018
Xanthoria parietina (L.) Th. Fr.	BP	8880.3	2019
Xanthoria parietina (L.) Th. Fr.	BP	8880.3	2019
Xanthoria parietina (L.) Th. Fr.	BP	8980.1	2019
Xanthoria parietina (L.) Th. Fr.	BP	8980.1	2019
Xanthoria parietina (L.) Th. Fr.	BP	8980.1	2019
Xanthoria parietina (L.) Th. Fr.	BP	8980.1	2019
Xanthoria parietina (L.) Th. Fr.	BP	8979.2	2019
Xanthoria parietina (L.) Th. Fr.	BP	8079.4	2020
Xanthoria parietina (L.) Th. Fr.	BP	8079.3	2020
Xanthoria parietina (L.) Th. Fr.	BP	8479.3	2020
Xanthoria parietina (L.) Th. Fr.	BP	8479.4	2020
Xanthoria parietina (L.) Th. Fr.	BP	9384.3	2020
Xanthoria parietina (L.) Th. Fr.	BP	9384.3	2020
Xanthoria parietina (L.) Th. Fr.	BP	8676.1	2021
Xanthoria parietina (L.) Th. Fr.	BP	8773.4	2021
Xanthoria parietina (L.) Th. Fr.	BP	8479.2	2021
Xanthoria parietina (L.) Th. Fr.	BP	8782.1	2021
Xanthoria parietina (L.) Th. Fr.	BP	8782.1	2021
Xanthoria parietina (L.) Th. Fr.	BP	8782.1	2021
Xanthoria parietina (L.) Th. Fr.	BP	8782.1	2021
Xanthoria parietina (L.) Th. Fr.	BP	9383.2	2021
Xanthoria parietina (L.) Th. Fr.	BP	8479.2	2021
Xanthoria parietina (L.) Th. Fr.	BP	0176.2	2021
Xanthoria parietina (L.) Th. Fr.	BP	8087.2	2021
Xanthoria parietina (L.) Th. Fr.	BP	8286.3	2021
Xanthoria parietina (L.) Th. Fr.	BP	9769.1	2021
Xanthoria parietina (L.) Th. Fr.	BP	8782.1	2021
Xanthoria parietina (L.) Th. Fr.	BP	8782.1	2021
Xanthoria parietina (L.) Th. Fr.	BP	8782.1	2021
Xanthoria parietina (L.) Th. Fr.	BP	9265.3	2021
Xanthoria parietina (L.) Th. Fr.	BP	9365.1	2021
Xanthoria parietina (L.) Th. Fr.	BP	9365.1	2021
Xanthoria parietina (L.) Th. Fr.	BP	9170.4	2022
Xanthoria parietina (L.) Th. Fr.	BP	9171.3	2022
Xanthoria parietina (L.) Th. Fr.	BP	8675.2	2022
Xanthoria parietina (L.) Th. Fr.	BP	8675.2	2022
Xanthoria parietina (L.) Th. Fr.	DE	7594.1	2022
Xanthoria parietina (L.) Th. Fr.	DE	7595.3	2022
Xanthoria parietina (L.) Th. Fr.	DE	8097.4	2022
Xanthoria parietina (L.) Th. Fr.	DE	8200.3	2022
Xanthoria parietina (L.) Th. Fr.	DE	8275.4	2022
Xanthoria parietina (L.) Th. Fr.	DE	8296.3	2022

Xanthoria parietina (L.) Th. Fr.	DE	8297.1	2022
Xanthoria parietina (L.) Th. Fr.	DE	8299.3	2022
Xanthoria parietina (L.) Th. Fr.	DE	8395.4	2022
Xanthoria parietina (L.) Th. Fr.	DE	8398.1	2022
Xanthoria parietina (L.) Th. Fr.	DE	8398.3	2022
Xanthoria parietina (L.) Th. Fr.	DE	8398.4	2022
Xanthoria parietina (L.) Th. Fr.	DE	8495.2	2022
Xanthoria parietina (L.) Th. Fr.	DE	8496.1	2022
Xanthoria parietina (L.) Th. Fr.	DE	8496.2	2022
Xanthoria parietina (L.) Th. Fr.	DE	8496.3	2022
Xanthoria parietina (L.) Th. Fr.	DE	8496.4	2022
Xanthoria parietina (L.) Th. Fr.	DE	8497.1	2022
Xanthoria parietina (L.) Th. Fr.	DE	8497.2	2022
Xanthoria parietina (L.) Th. Fr.	DE	8497.3	2022
Xanthoria parietina (L.) Th. Fr.	DE	8497.4	2022
Xanthoria parietina (L.) Th. Fr.	DE	8498.1	2022
Xanthoria parietina (L.) Th. Fr.	DE	8498.2	2022
Xanthoria parietina (L.) Th. Fr.	DE	8595.4	2022
Xanthoria parietina (L.) Th. Fr.	DE	8596.1	2022
Xanthoria parietina (L.) Th. Fr.	DE	8596.2	2022
Xanthoria parietina (L.) Th. Fr.	DE	8596.3	2022
Xanthoria parietina (L.) Th. Fr.	DE	8596.4	2022
Xanthoria parietina (L.) Th. Fr.	DE	8597.1	2022
Xanthoria parietina (L.) Th. Fr.	DE	8597.2	2022
Xanthoria parietina (L.) Th. Fr.	DE	8597.3	2022
Xanthoria parietina (L.) Th. Fr.	DE	8695.4	2022
Xanthoria parietina (L.) Th. Fr.	DE	8697.1	2022
Xanthoria parietina (L.) Th. Fr.	DE	8795.2	2022
Xanthoria parietina (L.) Th. Fr.	BP	9369.4	2022
Xanthoria parietina (L.) Th. Fr.	BP	9569.2	2022
Xanthoria parietina (L.) Th. Fr.	BP	8482.1	2022

[57] Niklfeld, H. Bericht über die Kartierung der Flora Mitteleuropa. *Taxon* **1971**, *20(4*), 545–571. https://doi.org/10.2307/1218258
[58] Borhidi, A. Role of mapping the flora of Europe in nature conservation. *Norrlinia* **1984**, *2*, 87–98.
